# Pan-Cancer Analysis of Immune Complement Signature C3/C5/C3AR1/C5AR1 in Association with Tumor Immune Evasion and Therapy Resistance

**DOI:** 10.3390/cancers13164124

**Published:** 2021-08-16

**Authors:** Bashir Lawal, Sung-Hui Tseng, Janet Olayemi Olugbodi, Sitthichai Iamsaard, Omotayo B. Ilesanmi, Mohamed H. Mahmoud, Sahar H. Ahmed, Gaber El-Saber Batiha, Alexander T. H. Wu

**Affiliations:** 1 PhD Program for Cancer Molecular Biology and Drug Discovery, College of Medical Science and Technology, Taipei Medical University and Academia Sinica, Taipei 11031, Taiwan; d621108004@tmu.edu.tw; 2 Graduate Institute for Cancer Biology & Drug Discovery, College of Medical Science and Technology, Taipei Medical University, Taipei 11031, Taiwan; 3 Department of Physical Medicine and Rehabilitation, Taipei Medical University Hospital, Taipei 11031, Taiwan; d301091012@tmu.edu.tw; 4 Department of Physical Medicine and Rehabilitation, School of Medicine, College of Medicine, Taipei Medical University, Taipei 11031, Taiwan; 5 Department of Medicine, Emory University School of Medicine, Atlanta, GA 30322, USA; janetola.olu@emory.edu; 6 Department of Anatomy, Faculty of Medicine and Research Institute for Human High Performance and Health Promotion (HHP&HP), Khon Kaen University, Khon Kaen 40002, Thailand; sittia@kku.ac.th; 7 Department of Biochemistry, Faculty of Science, Federal University Otuoke, Ogbia 23401, Bayelsa State, Nigeria; ilesanmiob@fuotuoke.edu.ng; 8 Department of Biochemistry, College of Science, King Saud University, Riyadh 11451, Saudi Arabia; mmahmoud2@ksu.edu.sa; 9 Medical Laboratory Technology Department, Faculty of Applied Medical Science, Misr University For Science &Technology, Cairo 3245310, Egypt; dr.sahar2010@hotmail.com; 10 Department of Pharmacology and Therapeutics, Faculty of Veterinary Medicine, Damanhour University, Damanhour 22511, AlBeheira, Egypt; gaberbatiha@gmail.com; 11 International Ph.D. Program for Translational Science, College of Medical Science and Technology, Taipei Medical University, Taipei 11031, Taiwan; 12 The PhD Program of Translational Medicine, College of Medical Science and Technology, Taipei Medical University, Taipei 11031, Taiwan; 13 Clinical Research Center, Taipei Medical University Hospital, Taipei Medical University, Taipei 11031, Taiwan; 14 Graduate Institute of Medical Sciences, National Defense Medical Center, Taipei 11490, Taiwan; 15 Taipei Heart Institute (THI), Taipei Medical University, Taipei 11031, Taiwan

**Keywords:** pan-cancer, tumor microenvironments, complement component proteins, tumor immune infiltrations, T-cell exclusion

## Abstract

**Simple Summary:**

We used multi-omics approaches to evaluate the association of complement signature C3/C5/C3AR1/C5AR1 with tumor immune phenotypes and prognosis across various cancer types. We found that the gene signatures have deregulated expression in human malignancies and demonstrated context-dependent association with tumor immune evasion, prognosis, and therapy response across the various cancer types. Further analysis revealed that *C3*, *C5*, *C3AR1*, and *C5AR1* were associated with tumor immune evasion via dysfunctional T-cell phenotypes with a lesser contribution of T-cell exclusion. Lastly, we also demonstrated that the expression levels of *C3*, *C5*, *C3AR1*, and *C5AR1* were associated with context-dependent chemotherapy, lymphocyte-mediated tumor killing, and immunotherapy outcomes in different cancer types. The complement components *C3*, *C5*, *C3AR1*, and *C5AR1* serve as attractive targets for strategizing cancer immunotherapy and response follow-up.

**Abstract:**

Despite the advances in our understanding of the genetic and immunological basis of cancer, cancer remains a major public health burden with an ever-increasing incidence rate globally. Nevertheless, increasing evidence suggests that the components of the complement system could regulate the tumor microenvironment (TME) to promote cancer progression, recurrence, and metastasis. In the present study, we used an integrative multi-omics analysis of clinical data to explore the relationships between the expression levels of and genetic and epigenetic alterations in *C3*, *C5*, *C3AR1*, and *C5AR1* and tumor immune evasion, therapy response, and patient prognosis in various cancer types. We found that the complements *C3*, *C5*, *C3AR1*, and *C5AR1* have deregulated expression in human malignancies and are associated with activation of immune-related oncogenic processes and poor prognosis of cancer patients. Furthermore, we found that the increased expression levels of *C3*, *C5*, *C3AR1*, and *C5AR1* were primarily predicted by copy number variation and gene methylation and were associated with dysfunctional T-cell phenotypes. Single nucleotide variation in the gene signature co-occurred with multiple oncogenic mutations and is associated with the progression of onco-immune-related diseases. Further correlation analysis revealed that *C3*, *C5*, *C3AR1*, and *C5AR1* were associated with tumor immune evasion via dysfunctional T-cell phenotypes with a lesser contribution of T-cell exclusion. Lastly, we also demonstrated that the expression levels of *C3*, *C5*, *C3AR1*, and *C5AR1* were associated with context-dependent chemotherapy, lymphocyte-mediated tumor killing, and immunotherapy outcomes in different cancer types. In conclusion, the complement components *C3*, *C5*, *C3AR1*, and *C5AR1* serve as attractive targets for strategizing cancer immunotherapy and response follow-up.

## 1. Introduction

Despite the advances in our understanding of the genetic and immunological basis of cancer, cancer remains a major public health burden with an ever-increasing incidence rate globally [1]. Nevertheless, increasing evidence suggests that the complement system and the tumor microenvironment (TME) play pivotal roles in cancer initiation, progression, recurrence, metastasis, and therapy failure [2]. The complement system is an important part of the innate immune system, which is essential for protection against infections and the removal of apoptotic cells. A complement identifies a foreign substance or injured cells and triggers a cascade of enzymatic events that mainly serves to stimulate phagocytosis by immune cells, inflammation of the surrounding tissue to attract additional phagocytes, and activation of the cell-killing membrane attack complex (MAC), which damages the cell membrane of the target cell via the formation of pores [3,4].

The complement system is classified into seven functional components: initiator complement components (e.g., the C1q complex, mannose-binding lectin (MBL)); enzymatic mediators (e.g., C1r, C1s, MASP2, and factor B, C3, and C5 convertases); membrane-binding components (e.g., C3b and C4b); inflammatory mediators: (e.g., C3a, C5a, and C4a); membrane attack proteins (e.g., C5b, C6, C7, C8, and C9); complement receptor proteins (e.g., CR1, C3aR1, and C5aR1); and regulatory complement components (e.g., factor I, factor H, CD59, and CD46). These proteins of the complement system are primarily in an inactivated form and are activated by a series of chain reaction pathways, the classical, lectin, and alternative pathways, which activate the complement components in sequential order. Inappropriate complement activations and dysregulation of the immune regulatory function of complements have been implicated in the development and progression of numerous inflammatory, degenerative, and autoimmune diseases [5,6,7,8].

However, activation of C3 and activation of C5 are major convergence points in the complement pathway [9] and, together with receptors, play a central and pivotal role in complement cascades [10,11]. C3 is the most abundant protein of the complement system and its receptor, C3aR, is widely distributed and is expressed on monocytes/macrophages, neutrophils, mast cells, and hepatocytes, among other cells [9]. C3aR and C5aR1 are anaphylatoxin receptors (ATRs) that belong to the superfamily of G-protein-coupled receptors, and their activation triggers a multitude of innate immune and inflammatory responses [12].

The binding of a complement component to receptors on neutrophils stimulates neutrophil degranulation and inflammation. C3aR1 and C5aR1 were found to induce Foxp3⁺ regulatory T cells and regulate tumorigenesis via C5a and C3a-dependent expression of TGF-β1, IL-6, and IL-10 [13]. Studies have also reported that complement C5 induces myeloid-derived suppressor cells (MDSCs) and suppresses the antitumor CD8+ T-cell response in TME [14,15]. C5, C3, C3aR1, and C5aR1 are therefore important in complement activities and have become leading therapeutic targets in many inflammatory diseases [10,11].

The TME comprises stromal cells (fibroblasts and immune cells), cancer cells, and extracellular components [16,17]. Cancer-associated fibroblasts (CAFs), MDSCs, tumor-associated macrophages (TAMs), regulatory T cells (Tregs), dendritic cells (DCs), and tumor-associated neutrophils (TANs) are communities of immunosuppressive cells that interact with cancer cells in the TME to enhance the tumor cells’ proliferation and invasion, malignant phenotypes, and the therapeutic response [18,19,20,21,22]. Complement proteins are abundant in the immune microenvironment [9] and emerging evidence indicates that the pathological activation of the complement system in the TME triggers tumorigenesis by regulating the inflammatory response and immunosuppressive stromal cells in the TME, thereby promoting the epithelial–mesenchymal transition (EMT), proliferation, migration, and tumor metastasis in various cancer types [23,24,25]. In agreement with the results of the current study, recent transcriptomic analysis of the complement genes’ expression and their clinical impact in different cancer types found context-dependent effects of complements on various cancer types [26]. Here, we used an integrative multi-omics analysis of clinical data to elucidate the association between the complement component (*C3*, *C5*, *C3AR1*, and *C5AR1*) and dysfunctional T-cell phenotypes, tumor immune evasion, therapy outcome, and prognosis in various cancer types, placing much emphasis on the effect of genetic and epigenetic alterations in the cancer-associated biological activities of the complement.

## 2. Materials and Methods

### 2.1. Pan-Cancer Analysis of Differential Gene Expression of C3, C5, C3AR1, and C5AR1 between Tumor and Normal Tissue, Tumor Stages, and Tumor Subtypes

We used the Tumor IMmune Estimation Resource (TIMER2.0) (http://timer.cistrome.org/ (accessed on 22 April 2021)) [27] to analyze the differential gene expression profile of *C3/C5/C3AR1/C5AR1* between adjacent normal tissue and tumor samples across the 33 TCGA cancer types. The list of the 33 TCGA cancer types, their histology, and their body location is provided in Table 1. We used GSCALite (http://bioinfo.life.hust.edu.cn/web/GSCALite/ (accessed on 26 April 2021)) [28], an integrated genomic and immunogenomic algorithm, to evaluate the differential expression of *C3*, *C5*, *C3AR1*, and *C5AR1* between the tumor stages (I, II, III, IV) and also between the tumor subtypes across 10,995 samples representing 33 TCGA cancer types (Table 1). The individual patient and sample IDs of each TCGA cancer type are available in the Supplementary Materials (File S1).

### 2.2. Pan-Cancer Analysis of Single Nucleotide Variations of C3, C5, C3AR1, and C5AR1

We collected TCGA SNV data and assessed the frequency and clinical effect of seven variant types of effective mutations (Missense_Mutation, Nonsense_Mutation, Frame_Shift_Ins, Splice_Site, Frame_Shift_Del, In_Frame_Del, In_Frame_Ins) of *C3*, *C5*, *C3AR1*, and *C5AR1* genes across 33 cancers from the NCI Genomic Data Commons (www.gdc.cancer.gov/ (accessed on 2 May 2021)). Only the SNVs of each gene’s coding region were considered, and we filtered out silent, Intron, IGR, 3′UTR, 5′UTR, 3′Flank, and 5′Flank SNVs. The percentage of SNVs of each gene’s coding region was calculated as %SNV = Num of Mutated Samples/Num of Cancer Samples [28]. We used Mutation Annotation Format (MAF) tools [29] for the visualization and summarization of the SNV data. In order to assess the relationship between gene set mutations and clinical outcomes of the patient, we used the R package to assess the overall survival differences between cohorts with mutated and cohorts with wild-type *C3/C5/C3AR1/C5AR1*. Cox regression hazards analysis of the cohorts in the mutated group and a log-rank test were conducted with a statistical significance cut-off (*p*-value < 0.05) [28]. In addition, we used the cBioPortal for Cancer Genomics (http://www.cbioportal.org/ (accessed on 5 May 2021)) to assess the gene mutation co-occurrence pattern between the *C3/C5/C3AR1/C5AR1* signature and other gene mutations across 10,953 patients from TCGA Pan-Cancer Atlas Studies [30].

### 2.3. Pan-Cancer Methylation Analysis of C3, C5, C3AR1, and C5AR1

We used the methylation module of the GSCALite algorithm [28] to analyze the differential methylation levels of *C3*, *C5*, *C3AR1*, and *C5AR1* between tumor and paired normal tissues across the cancer types. The methylation difference between tumor and normal samples was compared using Student’s *t*-test at a FDR-adjusted *p*-value ≤ 0.05. In addition, we analyzed the effect of the methylation on gene expression by assessing the correlation between the mRNA expression and methylation levels of each gene based on Pearson’s correlation coefficient and the t distribution with a FDR-adjusted *p*-value [31]. A log-rank test was used to compare the survival differences between cohorts with hypomethylated genes and cohorts with hypermethylated genes. *p*-values were considered significant at <0.05. Cox regression [32] was performed to estimate the hazards. *p*-values < 0.05 were considered significant.

### 2.4. Pan-Cancer Analysis of Copy Number Variation in C3, C5, C3AR1, and C5AR1

The frequency of four types of copy number variation (CNV), including the Hete Amp: heterozygous amplification (CNV = 1), Hete Del: heterozygous deletion (CNV = −1), Homo Amp: homozygous amplification (CNV = 2), and Homo Del: homozygous deletion (CNV = −2) of *C3*, *C5*, *C3AR1*, and *C5AR1* was assessed across the 33 cancer types (Table 1). The CNV data were processed with GISTICS2.0 [33]. In addition, we analyzed the association between mRNA expression and CNV based on Pearson’s product-moment correlation coefficient and the t distribution. *p*-values were adjusted by FDR (*p* ≤ 0.05).

### 2.5. Pan-Cancer Analysis of the C3, C5, C3AR1, and C5AR1 Association with Tumor Immune and Immune-Suppressive Cell Infiltrations, Dysfunctional T-Cell Phenotype, and T-Cell Exclusion

The correlation of *C3*, *C5*, *C3AR1*, and *C5AR1* expression with tumor infiltrations of six types of immune cells (B cells, CD8+ T cells, CD4+ T cells, macrophages, neutrophils, and dendritic cells) was analyzed using the TIMER2.0 algorithm. The raw file of the immune infiltration estimation for all TCGA tumors used in this study can be found in the Supplementary Materials (File S2). In addition, we used the ImmuCellAI (immune cell abundance identifier), algorithm (http://bioinfo.life.hust.edu.cn/ImmuCellAI#!/ (accessed on 9 July 2021)), a signature-based gene set for estimating the tumor infiltration of immune cells, to analyze the correlation between the *C3*, *C5*, *C3AR1*, and *C5AR1* expression levels and the abundance of the immune cells across the TCGA cancer types (File S3). The correlation analysis was conducted using the purity-corrected partial Spearman’s rho value and statistical significance based on the *p*-value of a Wilcoxon test and FDR. Data analysis and visualization were done using the GSCALite online server [28]. We used the TIMER and TIDE algorithms to evaluate the effect of *C3*, *C5*, *C3AR1*, and *C5AR1* genes on T-cell exclusion by examining the correlation between the gene expression levels and tumor infiltration of three cell types reported to restrict T-cell infiltration in tumors, namely cancer-associated fibroblasts (CAFs), myeloid-derived suppressor cells (MDSCs), and the M2 subtype of tumor-associated macrophages (TAMs) [34]. Furthermore, we explored the association between the CNV of the gene signature, the methylation level of the gene signature, and dysfunctional T-cell phenotypes using the QUERY module of the TIDE Server (http://tide.dfci.harvard.edu/query/(accessed on 10 May 2021)) [35]. The source code used for the computation of the T-cell dysfunction score can be accessed via GitHub (https://github.com/foreverdream2/dysfunction_interaction_test/releases (accessed on 10 May 2021)) [36].

### 2.6. Functional Enrichment and PPI Network Analysis

We used the Enrichr (https://maayanlab.cloud/Enrichr/enrich# (accessed on 21 May 2021)) web server for the Kyoto Encyclopedia of Gene and Genome (KEGG) enrichment analyses of *C3*, *C5*, *C3AR1*, and *C5AR1* [37,38]. Furthermore, we integrated the complement genes with their co-occurring mutated genes and performed the KEGG and gene ontology (GO) enrichment analyses using the ToppGene Suite (http://toppgene.cchmc.org (accessed on 21 May 2021)) algorithm at default parameters. We used the “Bonferroni” multiple correction method and a *p*-value of 0.01 for the significance cut-off level [39,40].

### 2.7. Analysis of Gene Expression Correlation with Drug Sensitivity and Immunotherapy Response

Following the protocol described by Rees et al. [41], we used the GSCALite server to download the area under the dose–response curve (AUC) values for drugs and gene expression profiles of C3, C5, C5AR1, and C3AR1 in human cancer cell lines from the Genomics of Drug Sensitivity in Cancer (GDSC) database, a database containing data on the drug sensitivity of well-characterized human cell lines. We then used the Spearman method to analyze the correlation between the gene expression levels of *C3*, *C5*, *C5AR1*, and *C3AR1*, drug sensitivity (IC_50_), and 265 small molecules. In addition, the ROC plotter tool (http://www.rocplot.org/ (accessed on 25 May 2021)) was utilized to analyze the association between the gene expression level and response to therapy; this analysis was based on the transcriptome-level data from patients with breast, glioblastoma, colorectal, and ovarian cancer [42]. Lastly, we used the TIDE algorithm (http://tide.dfci.harvard.edu/ (accessed on 21 May 2021)) to evaluate the immunotherapy outcome of cancer patients following treatment with immune checkpoint blockade (ICB) as measured by the survival differences between patients with high gene expression levels and patients with low gene expression levels [36].

### 2.8. Gene Prioritization of C3, C5, C3AR1, and C5AR1 across Four Immunosuppressive Indices

In order to identify potential gene targets, we used the regulator prioritization module of the TIDE algorithm to rank the importance of the genes based on the dysfunction and risk scores computed from clinical studies and CRISPR screening processes. We assessed the gene prioritization of *C3*, *C5*, *C3AR1*, and *C5AR1* across four immunosuppressive parameters, including T-cell exclusion score, T-cell dysfunction score, response to immune checkpoint blockade (ICB) therapy, and gene knockout phenotype in CRISPR screens. The T-cell dysfunction score was used to evaluate how C3, C5, C5AR1, and C3AR1 interact with cytotoxic T cells to influence the survival of cancer patients, while the T-cell exclusion score was used to evaluate the collective effect of three immunosuppressive cell types (CAF, MDSCs, and M2-TAMs) on T-cell exclusion in the TME. The z-score in the Cox PH regression was used to evaluate the effect of the gene expression on patient survival in ICB treatment cohorts. The normalized logFC in CRISPR screens was employed in the evaluation of the effect of gene-knockout-mediated and lymphocyte-induced tumor death in cancer models [35].

### 2.9. Comparative Biomarker Evaluation between Standardized Biomarkers and the C3, C5, C3AR1 and C5AR1 Gene Set

Subsequently, we used the custom Geneset Prediction Function and Biomarker Evaluation Module of the TIDE server [35] to customize the *C3*, *C5*, *C3AR1*, and *C5AR1* genes and compare their prognostic relevance to that of standardized biomarkers, including the T-cell clonality assessment (T. Clonality), the B-cell clonality assessment (B. Clonality), TIDE, the estimating microsatellite instability (MSI) score, the tumor mutational burden (TMB), cluster of differentiation 274 (CD274), and interferon gamma (IFNG), based on their predictive power with respect to response outcome and overall survival in different cancer types [35,36].

### 2.10. Gene Pathway Activity and Interaction Network

The reverse-phase protein array (RPPA) data from The Cancer Proteome Atlas (TCPA) database (https://www.tcpaportal.org/tcpa/ (accessed on 5 May 2021)) were used to calculate the pathway activity score of the complement components C3, C5, C3AR1, and C5AR1 in activating or inhibiting 10 famous cancer-associated signaling pathways, including the apoptosis, TSC/mTOR, EMT, RTK, hormone ER, RAS/MAPK, hormone AR, DNA damage response, cell cycle, and PI3K/AKT signaling pathways, in 32 TCGA cancer types according to the method described by Akbani et al. [43]. The RPPA data were median-centered and normalized by the standard deviation across all samples for each component to obtain the relative protein level. The pathway score was then calculated as the sum of the relative protein level of all positive regulatory components minus that of all negative regulatory components on a particular pathway. The expression and pathway activity module of the GSClite algorithm was used to construct the gene pathway activity and interaction network and the heat map of the percentage of cancers in which a gene has an effect (activation or inhibition) on the pathway among the selected cancer types.

### 2.11. Data Analysis and Visualization

We used GraphPad Prism software (version 8.0.0 for Windows) for data visualization. The differentially expressed genes between tumor and normal samples were compared using the Wilcoxon test. Heat maps were used to visualize the infiltration levels of the immune cells across the 33 TCGA cancer types. A bubble plot and a Kaplan–Meier plot were used for the visualization of survival differences between cohort groups. All values were considered statistically significant at *: a *p*-value < 0.05; **: a *p*-value < 0.01; ***: a *p*-value < 0.001.

## 3. Results

### 3.1. Complement Components C3, C5, C3AR1, and C5AR1 Demonstrated Context-Dependent Deregulatory Expression and Are Associated with Activation of Immune-Related Oncogenic Processes and Prognosis of the Cohort in Various Cancer Types

**Figure 1:**
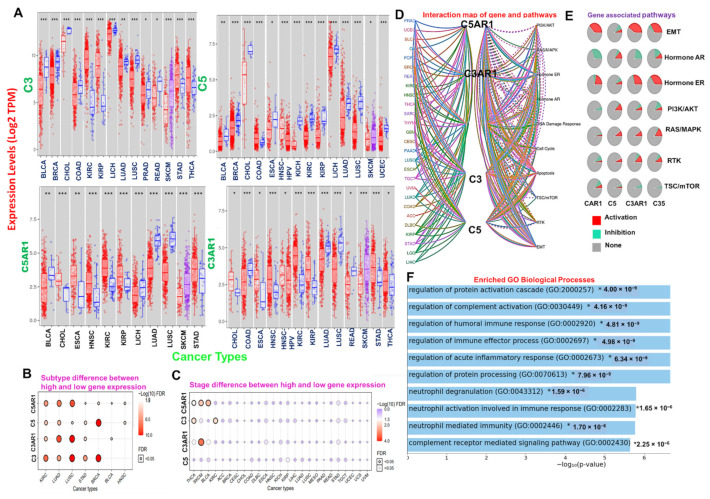
Differential expression of *C3*, *C5*, *C3AR1*, and *C5AR1* in human malignancies is associated with activation of immune-related oncogenic processes and poor prognosis of cancer patients. (**A**) Comparative *C3*, *C5*, *C3AR1*, and *C5AR1* expression profiles between tumor and adjacent normal samples across the TCGA database. The blue and red plots represent the tumor and normal sample, respectively. A heat map showing the difference in gene expression between (**B**) the tumor subtypes and (**C**) TNM stages across the TCGA database. The color from white to red represents the FDR significance. The bubble color from blue to red and the size represent the FDR significance. The black outline border indicates FDR < 0.05. (**D**) Interaction map of genes and pathways. *C3*, *C5*, *C3AR1*, and *C5AR1* (**E**) associated pathways (**F**) and enriched biological processes. *: *p*-value < 0.05; **: *p*-value < 0.01; ***: *p*-value < 0.001.

We used the differential gene expression module of TIMER to evaluate the differential expression of *C3*, *C5*, *C3AR1*, and *C5AR1* between human cancer and normal tissues across the TCGA database. Our results indicate that C3 is downregulated in BLCA, BRCA, CHOL, COAD, LIHC, LUAD, LUSC, PRAD, and READ but upregulated in KIRC, KIRP, SKCM metastasis, STAD, and THCA; C5 is downregulated in BLCA, BRCA, CHOL, ESCA, KICH, KIRC, KIRP, LIHC, LUAD, LUSC, SKCM, THCA, and UCEC but upregulated in COAD and HNSC-HPVpos; C5AR1 is downregulated in BLCA, LIHC, LUAD, and LUSC but upregulated in CHOL, ESCA, HNSC, KIRC, KIRP, SKCM metastasis, and STAD; and C3AR1 is downregulated in COAD, LUAD, LUSC, and READ but upregulated in ESCA, HNSC-HPVpos, HNSC, KIRC, KIRP, CHOL, SKCM, STAD, and THCA when compared with the adjacent normal tissue (Figure 1A). Analysis of differential gene expression at different tumor stages revealed an association between tumor stages and high gene expression of C3 in THCA and KIRC; C3AR1 in SKCM; and C5AR1 in BLCA, THCA, and SKCM (Figure 1B). Furthermore, high expression levels of *C3*, *C5*, *C3AR1*, and *C5AR1* are significantly (*p* < 0.05) associated with tumor subtypes in KIRC, LUAD, LUSC, and STAD, while tumor stages of BRCA are significantly (*p* < 0.05) associated with the expression of C3, C5, and C5AR1 (Figure 1C).

We conducted a gene pathway activity and interaction network analysis to identify the effect of the complement component genes *C3*, *C5*, *C3AR1*, and *C5AR1* on 10 major functional and signaling pathways associated with human cancer. Our results reveal that these genes were greatly enriched in the activation of oncogenic pathways, including EMT, ER-hormonal signaling, PI3K/AKT, RAS/MAPK, RTK, and mTOR (Figure 1D,E). In addition, several biological and immune-related processes, including regulation of protein activation cascade, neutrophil activation, acute inflammatory response, complement receptor-mediated signaling pathway, regulation of humoral immune response, and immune effector processes, were enriched in the gene signature (Figure 1F). To evaluate the prognostic relevance of the gene signature, we assessed the association between the gene expression levels and the overall or progression-free survival of the cohort. We found that higher expression levels of C3 in COAD, GBM, KIRC, LGG, and LUSC; C3AR1 in GBM, LGG, and COAD; C5 in COAD, KICH, STAD, and UVM; and C5AR1 in THCA tumors are associated with a shorter survival duration of the cohorts. In contrast, a longer survival duration of the cohorts was achieved with higher expression levels of C3 in ACC, MESO, and SKCM; C3AR1 in ACC and SKCM; C5 in LICH, SKCM, and UCEC; and C5AR1 in DLBC (Table S1).

### 3.2. C3, C5, C3AR1, and C5AR1 Are Associated with Context-Dependent Tumor Immune Evasion via Dysfunctional T-Cell Phenotypes with a Lesser Contribution of T-Cell Exclusion

**Figure 2:**
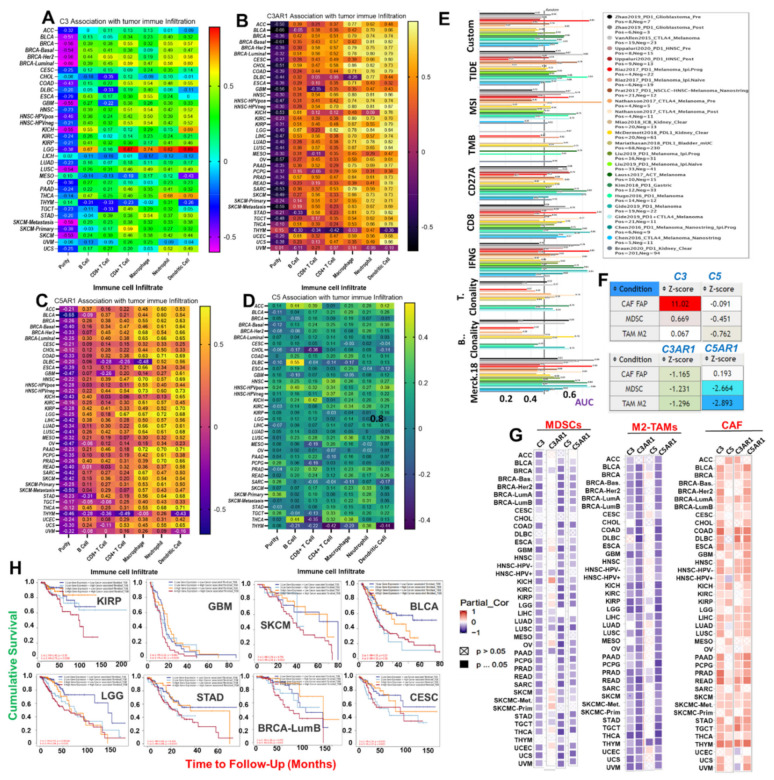
*C3*, *C5*, *C3AR1*, and *C5AR1* are associated with tumor immune evasion via dysfunctional T-cell phenotypes with a lesser contribution of T-cell exclusion. A heat map showing the correlation of (**A**) C3, (**B**) C3AR1 (**C**) C5AR1, and (**D**) C5 expression with immune infiltration level in diverse cancer types. The correlation is depicted with the purity-corrected partial Spearman’s rho value and statistical significance. (**E**) Bar plot showing the biomarker significance of *C3/C5/C3AR1/C5AR1* in comparison with standardized cancer immune evasion biomarkers in an ICB sub-cohort. The area under the receiver operating characteristic curve (AUC) was applied to evaluate the prediction performance of the tested biomarkers on the ICB response status. The (**F**) TIDE score and (**G**) heat map of the correlation between gene expression and infiltrations of cancer-associated fibroblasts (CAFs), myeloid-derived suppressor cells (MDSCs), and the M2 subtype of tumor-associated macrophages (TAMs) in different cancer types. (**H**) Kaplan–Meier plot of the cumulative survival of cancer cohorts with different degrees of CAF infiltration and *C3*, *C5*, *C3AR1*, and *C5AR1* expression levels.

We assessed the correlation of *C3*, C5, C3AR1, and C5AR1 expression with infiltrations of six types of immune cells across the TCGA cancer types. C3 expression shows a very strong correlation (*r* > 0.7) with the infiltration of various immune cells in LGG and only dendritic cells in THCA. C3 expression is strongly (*r* = 0.5~0.7) associated with the infiltration of various immune cells, including B-cells in KICH; CD8+ T cells in BRCA-Her2; CD4+ T cells in BRCA-Her2, BRCA-Luminal, SKCM-Primary, HNSC, HNSC-HPVneg, and COAD; macrophages in COAD, ESCA, HNSC, HNSC-HPV_POS,_ HNSC-HPVneg, and STAD; neutrophils in THCA, BRCA, BRCA-Her2, BRCA-Luminal, and UCS; and dendritic cells in BRCA, BRCA-Her2, COAD, HNSC, HNSC-HPV_POS_, HNSC-HPVneg, STAD, BRCA-Luminal, and KIRC (Figure 2A). C3AR1 shows a very strong correlation (*r* > 0.7) with the infiltration of dendritic cells in 23 types of TCGA cancers, and a strong correlation (*r* = 0.5~<0.7) with the infiltration of dendritic cells in five cancer types (SKCM-Metastasis, UCEC, OV, TGCT, and BLCA). Neutrophil infiltrations correlate very strongly (*r* > 0.7) with C3AR1 in 17 cancer types and strongly in 13 cancer types; macrophage infiltrations correlate very strongly (*r* > 0.7) with C3AR1 in 16 cancer types and correlate strongly (*r* = 0.5~<0.7) with C3AR1 in six cancer types (SKCM, SKCM-Metastasis, STAD, PRAD, LUSC, and KIRP); and B-cell infiltrations correlate strongly (*r* = 0.5~<0.7) with C3AR1 in 11 cancer types (UCEC, LICH, THCA, BRCA-Luminal, CHOL, KICH, KIRP, LGG, PRAD, THCA, and THYM). Similarly, CD8+ T-cell infiltrations correlate strongly (*r* = 0.5~<0.7) with C3AR1 in 11 cancer types (SKCM, SKCM-Primary, SKCM-Metastasis, STAD, BRCA, HNSC, HNSC-HPVneg, LUSC, PAAD, BRCA-Her2, and BRCA-Luminal). CD4+ T-cell infiltrations show a very strong correlation (*r* > 0.7) with C3AR1 in HNSC-HPVneg and LGG, and a strong correlation (*r* = 0.5~<0.7) with C3AR1 in CHOL, BRCA, HNSC, BRCA-Basal, BRCA-Her2, and BRCA-LuminalB (Figure 2B).

C5AR1 expression correlates very strongly (*r* > 0.7) with the infiltration of macrophages in HNSC, HNSC-HPVneg, and PAAD; neutrophils in PAAD, PRAD, COAD, and LGG; and dendritic cells in THCA, PAAD, KIRP, and HNSC-HPVneg. C5AR1 expression correlates strongly (*r* > 0.7) with the infiltration of macrophages in HNSC-HPVneg, LIHC, SKCM-Primary, SKCM-Metastasis, STAD, and THCA; neutrophils in PCPG, LUSC, SKCM, STAD, THCA, LIHC, LUAD, KIRP, KIRC, HNSC, DLBC, CHOL, and BRCA-Her2; and dendritic cells in various cancer types (ACC, BLCA, BRCA, BRCA-Basal, BRCA-Her2, BRCA-Luminal COAD, DLBC, GBM, HNSC, KICH, LGG, LIHC, LUAD, LUSC, MESO, PRAD, READ, SARC, SKCM, STAD, and UCS). However, C5AR1 expression shows a weak (*r* = 0.2~<0.5) to poor (*r* < 0.2) correlation with tumor infiltrations of CD4+ T cells, CD4+ T cells, and B cells in various cancer types (Figure 2C). Similarly, C5 shows weak (*r* = 0.2~<0.5) or poor correlation (*r* < 0.2) with the infiltration of all six types of immune cells in all 33 TCGA cancer types (Table 1, Figure 2D). Interestingly, the levels of correlation between the expression levels of the complement component genes and the immune infiltration were consistent with the data generated using ImmuCellAI (immune cell abundance identifier) algorithms (File S3).

Furthermore, we evaluated the prognostic relevance of the gene signature by comparison with standardized biomarkers based on their predictive power with respect to response outcome and overall survival in ICB sub-cohorts. Interestingly, we found that the gene signatures of *C3*, *C5*, *C3AR1*, and *C5AR1* gave an area under the receiver operating characteristic curve (AUC) value greater than 0.5 in 12 out of the 23 ICB sub-cohorts (Figure 2E). Compared with the gene signature, TMB, T.Clonality, B.Clonality, and MSI SCORE demonstrated lower biomarker relevance in ICB sub-cohorts. However, the predictive power of the signature is lower than that of TIDE, CD274, and IFNG as biomarkers in ICB sub-cohorts (Figure 2E). We also assessed the correlation between gene expression and infiltrations of T-cell exclusion signatures, including CAF, M2-TAMs, and MDSCs. We found that *C3*, *C5*, *C3AR1*, and *C5AR1* expression is negatively associated with infiltrations of M2-TAMs and MDSCs (Table S2) but positively associated with CAF infiltrations in various cancer types (Figure 2F,G). Interestingly, the association of a high degree of CAF infiltration with a high gene expression level also showed a significant association with poor survival of KIRP, GBM, SKCM, BLCA, LGG, STAD, CESC, and BRCA-Lumb cohorts (Figure 2H). Collectively, these correlation analyses established a strong association between *C3*, *C5*, *C3AR1*, and *C5AR1* and tumor immune evasion via dysfunctional T-cell phenotypes with a less significant contribution from T-cell exclusion.

### 3.3. SNVs of C3, C5, C3AR1, and C5AR1 Are Associated with Prognosis and Co-Occurred with Other Oncogenic Mutations

**Figure 3:**
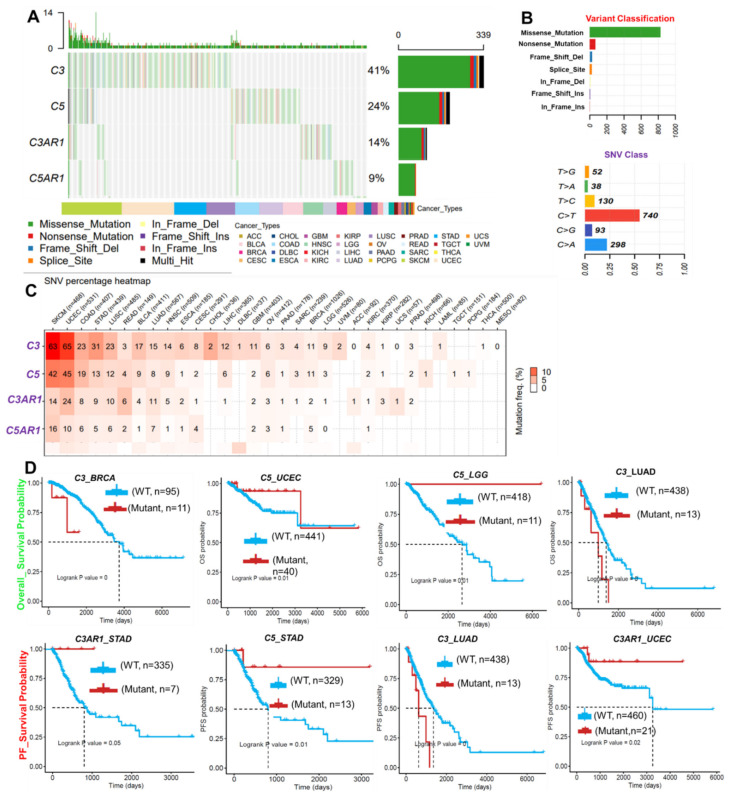
SNV in *C3*, *C5*, *C3AR1*, and *C5AR1* is associated with prognosis and co-occurred with other oncogenic mutations (**A**) The waterfall plot showing the mutation distribution and SNV classification of SNV types in *C3*, *C5*, *C3AR1*, and *C5AR1*. (**B**) The summary plot of the SNV variant classification of *C3*, *C5*, *C3AR1*, and *C5AR1* across the TCGA database. (**C**) Heat map showing the SNV frequency of *C3*, *C5*, *C3AR1*, and *C5AR1* across the cancer types. (**D**) Kaplan–Meier plot of the overall survival and progression-free survival of cohorts with mutant or wild-type *C3*, *C5*, *C3AR1*, and *C5AR1*.

Our analysis of the single nucleotide variation in the gene signature revealed that *C3*, *C5*, *C3AR1*, and *C5AR1* have mutation frequencies of 41%, 24%, 14%, and 9%, respectively, across the TCGA cancer types (Figure 3A, Table S3). According to the variant classification, a missense mutation is the most prevalent SNV of *C3*, *C5*, *C3AR1*, and *C5AR1* in TCGA cancer cohorts (Figure 3B). Specifically, the majority of the *C3*, *C5*, *C3AR1*, and *C5AR1* mutations transitioned, including the C > T and T > C transitions followed by the C > A transversion (Figure 3B). Among the gene signatures, C3 and C5 are the most frequently mutated, while C3AR1 and C5AR1 are less frequently mutated across the cancer types. However, according to the cancer types, SNV occurs most frequently in the order of SKCM, UCEC, COAD, STAD, HNSC, LUSC, BLCA, LUAD, ESCA, and BRCA (Figure 3C). Survival analysis of the cohorts revealed that SNV in C3 is associated with shorter overall and progression-free survival of the BRCA and LUAD cohorts. SNV in C5 is associated with prolonged survival of the LGG, LUAD, STAD, and UCEC cohorts, while SNV in C3AR1 also prolonged the lifespan of the STAD and UCEC cohorts (Figure 3D, Figure S1). Furthermore, we found that the SNV in *C3*, *C5*, *C3AR1*, and *C5AR1* co-occurred significantly with mutation of a number of oncogenic proteins (Figure S2, Table S4) associated with several immune and oncogenic-related pathways, biological processes, and onco-immune-related diseases (Table 2).

### 3.4. C3, C5, C3AR1, and C5AR1 Expression Exhibited a Tumor-Context-Dependent Association with Copy Number Variation, Gene Methylation, and Dysfunctional T-Cell Phenotypes

**Figure 4:**
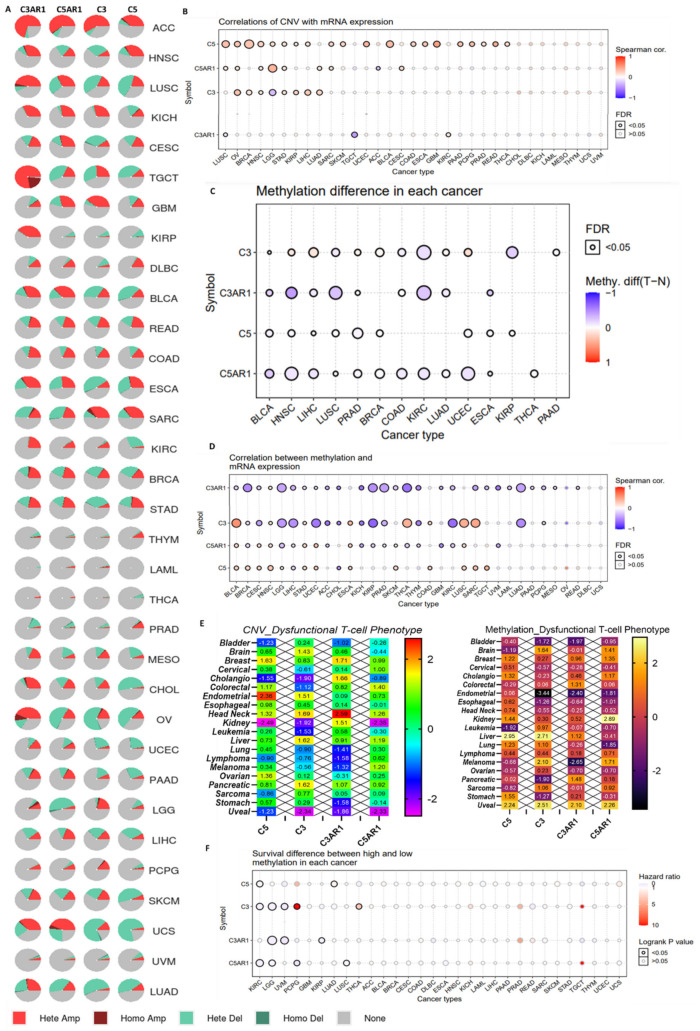
CNV and gene methylation are associated with the expression of *C3*, *C5*, *C3AR1*, and *C5AR1* and dysfunctional T-cell phenotypes. (**A**) Pie chart showing the distribution of the CNVs of CE/C5/C3AR1/C5AR1 across TCGA cancer types. Hete Del: heterozygous deletion; Hete Amp: heterozygous amplification; Homo Del: homozygous deletion; Homo Amp: homozygous amplification. (**B**) Correlation of CNVs with mRNA expression of *C3*, *C5*, *C3AR1*, and *C5AR1* in different cancer types. (**C**) Bubble plot of the methylation differences between tumor and normal samples. (**D**) Correlation of methylation with mRNA expression in different cancers. The blue and red bubbles represent a negative and positive correlation, respectively. The deeper the color, the higher the correlation. The size of the point represents statistical significance; the bigger the size, the more statistically significant. (**E**) Heat map of the association between dysfunctional T-cell phenotypes and CNVs (left panel) or methylation (right panel) of CE/C5/C3AR1/C5AR1 across different TCGA cancer types. (**F**) Bubble plot of the survival differences between cohorts with hypermethylation and cohorts with hypomethylation of *C3*, *C5*, *C3AR1*, and *C5AR1* in different cancer types. The blue points represent patients with hypomethylated genes having worse survival, while red points represent patients with hypermethylated genes having worse survival.

Somatic copy number alterations (SCNAs) are widespread in human cancers and have been suggested to drive tumorigenesis. Whether and how tumor SCNA levels influence immune evasion is of particular interest. We explored the relationships between CNAs of the complement component and the tumor immune microenvironment by examining the correlations between CNAs of *C3 C5*, C3AR1, C5AR1, and dysfunctional T-cell phenotypes in TCGA cancer cohorts. We found that CNVs of *C3*, C5, C3AR1, and *C5AR1* occur in all 33 TCGA cancer types analyzed and at a high frequency in ACC, HNSC, LUSC, KICH, CESC, TGCT, BLCA, READ, ESCA, SARC, OV, UCEC, SKCM, LUAD, and UCS, while CNVs of *C3*, C5, C3AR1, and *C5AR1* occur less frequently in THYM, LAML, THCA, and PRAD. By CNV stratification, gene heterozygous amplification and heterozygous deletion are the most frequently occurring CNVs, while homozygous amplification and deletion occur less frequently (Figure 4A). Correlation analysis revealed a positive correlation between mRNA expression and CNVs of C5 in LUSC, OV, BRCA, HNSC, LGG, STAD, KIRP, LICH, LUAD, SARC, SKCM, UCEC, BLCA, COAD, ESCA, GBM, PAAD, PCPG, PRAD, READ, and THCA; CNVs of C5AR1 in LUSC, OV, HNSC, LGG, STAD, LUAD, SARC, and CESC; CNVs of C3 in OV, BRCA, HNSC, STAD, KIRP, LICH, and LUAD; and CNVs of C3AR1 in KIRC. We observed a negative correlation between mRNA expression and CNVs of C3 in LGG; C3AR1 in TGCT; and C5AR1 in ACC (Figure 4B). Furthermore, we found that C5, C3, C5AR1, and C3AR1 are hypomethylated (Figure 4C) and negatively correlated with the mRNA expression patterns in multiple cancers. However, methylation of C3 shows a positive correlation with the mRNA expression levels in BLCA, THCA, LUSC, and SARC, while C5 methylation is positively correlated with BLCA, BRCA, CESC, HNSC, STAT, UCEC, SKCM, COAD, LUSC, SARC, and TGCT (Figure 4D).

We queried the effect of CNVs and differential methylation levels of *C3/C5/C3AR1/C5AR1* on T-cell dysfunction phenotypes across the TCGA cancer types. Interestingly, we found a strong association between T-cell dysfunction phenotypes and CNVs of C5 in breast, colorectal, endometrial, head and neck, and ovarian cancer; CNVs of C3 in brain, endometrial, head and neck, liver, and pancreatic cancer; CNVs of C3AR1 in breast, cholangitis, kidney, head and neck, and pancreatic cancer; and CNVs of C5AR1 in colorectal, head and neck, liver, and melanoma cancer. Furthermore, the T-cell dysfunction phenotype is associated with differential methylation levels of C5 in brain, breast, cholangio, kidney, leukemia, liver, lung, stomach, and uveal cancer; C3 in bladder, brain, endometrial, esophageal, liver, lung, melanoma, pancreatic, sarcoma, stomach, and uveal cancer; and C3AR1 in bladder, colorectal, endometrial, liver, melanoma, pancreatic, and uveal cancer. Differential methylation of C5AR1 is associated with T-cell dysfunction phenotypes in brain, breast, cholangio, endometrial, esophageal, kidney, lung, melanoma, and uveal cancer (Figure 4E). Survival analysis indicated that cohorts with hypomethylation of C5 in KIRC; C3 in KIRC, LGG, and UVM; C3AR1 in LGG, UVM, and KIRP; and C5AR1 in KIRC, LGG, PCPG, and LUSC had a poor prognosis. However, hypermethylation of C3 achieved a poor prognosis in the PCPG and THCA cohorts (Figure 4F).

### 3.5. C3, C5, C3AR1, and C5AR1 Are Associated with Chemotherapy Outcome in Multiple Cancer Types

**Figure 5:**
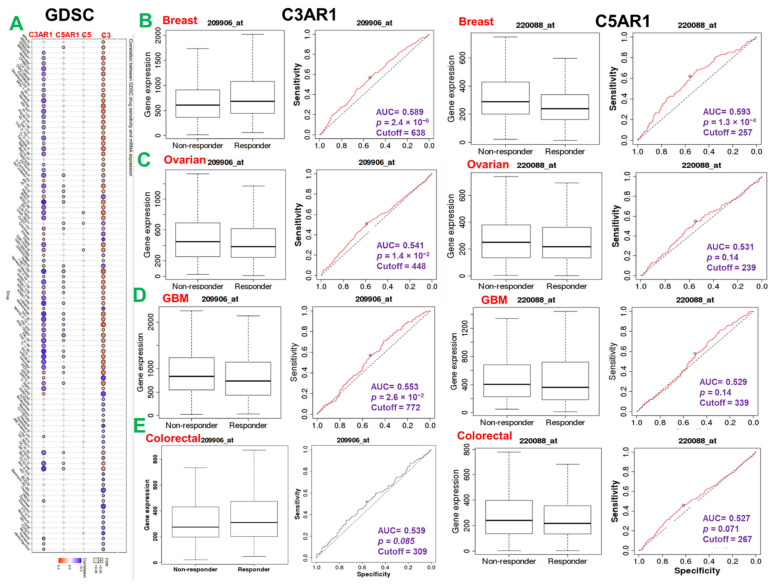
*C3*, *C5*, *C3AR1*, and *C5AR1* expression is associated with response to chemotherapy in multiple cancer types. (**A**) Bubble plot of the correlation between GDSC drug sensitivity and mRNA expression of C3, C5, C5AR1, and C3AR1. The color from blue to red represents the correlation between mRNA expression and IC50. A positive correlation means that a gene with a high level of expression is resistant to the drug and vice versa. The bubble size positively correlates with the FDR significance. The black outline border indicates FDR < 0.05. The ROC plot of the association between C3AR1/C5ARI expression and the response to therapy of brain (**D**), breast (**B**), ovarian (**C**), and colorectal cancer (**E**).

Genetic alterations influence the drug sensitivity of cancers to clinical therapies and are potential biomarkers for drug screening. Therefore, we queried the association between the mRNA expression levels of C3, C5, C5AR1, and C3AR1 and the sensitivity of patients to chemotherapy and immunotherapy. Interestingly, we found that high expression of C3 and low expression of C3AR1 are associated with resistance to chemotherapy for several GDSC small molecules (Figure 5A). Specifically, we found that low expression levels of C3AR1 are associated with resistance to chemotherapy in colorectal and breast cancer but increased the drug sensitivity in GBM and ovarian cancer cohorts. High expression levels of C5AR1, on the other hand, are significantly associated with resistance to chemotherapy in the breast, ovarian, colorectal, and GBM cohorts (Figure 5B).

### 3.6. C3, C5, C3AR1, and C5AR1 Are Associated with Lymphocyte-Mediated Tumor Killing and Immunotherapy Outcome

**Figure 6:**
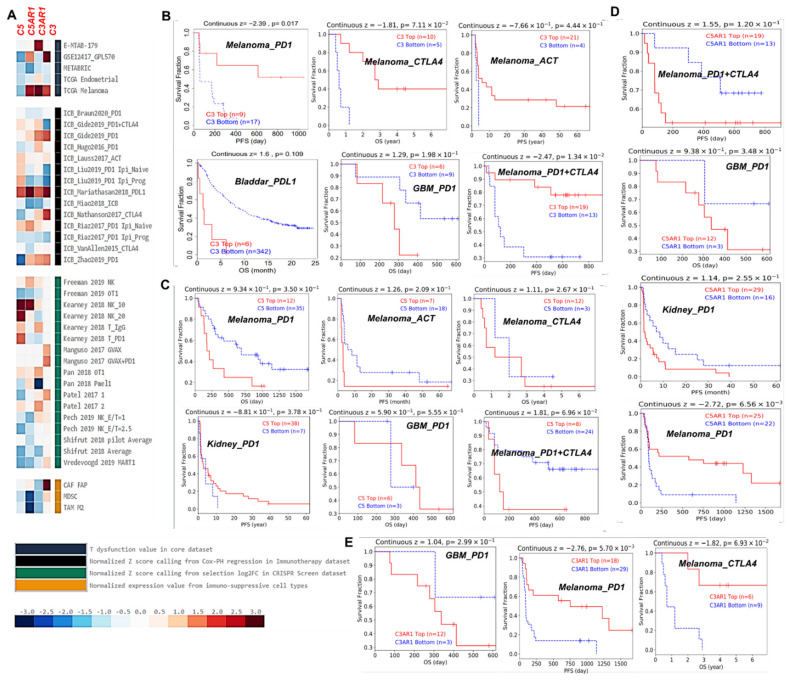
*C3*, *C5*, *C3AR1*, and *C5AR1* are associated with lymphocyte-mediated tumor killing and immunotherapy outcome (**A**) Prioritization of *C3*, *C5*, *C3AR1*, and *C5AR1* across four immunosuppressive indices, including T-cell exclusion, T-cell dysfunction phenotypes, association with immune therapy outcome, and lymphocyte-mediated tumor killing in CRISPR screens. Kaplan–Meier plot of the survival differences between ICB-treated cohorts with high gene expression and cohorts with low gene expression of (**B**) C3, (**C**) C5, (**D**) C5AR1, and (**E**) C3AR1.

Finally, we assessed the gene prioritization of *C3*, *C5*, *C3AR1*, and *C5AR1* in order to summarize the role of each gene association with four immunosuppressive indices, including the ICB response outcome, T-cell dysfunction levels, T-cell exclusion levels, and phenotypes in genetic screens (CRISPR screens), in a range of cohorts. We found that C3, C3AR1, and C5AR1 are associated with T-cell dysfunction phenotypes in two (GSE12417_GPL570 and TCGA melanoma) of the five datasets enumerated (Figure 6A, upper panel). C3AR1 appears to be of higher priority as it is associated with T-cell dysfunction phenotypes in three of the four datasets enumerated, while C5 is of lower priority as it shows a negative association with T-cell dysfunction phenotypes in all four datasets enumerated (Figure 6A, upper panel). Meanwhile, high expression of *C3*, *C5*, *C3AR1*, and *C5AR1* is associated with worse PDL1 outcomes in bladder cancer (ICB_Mariathasan2018_PDL1) and PD1 (ICB_Gide2019_PD1) and ACT (ICB_Lauss2017_ACT) outcomes in melanoma and treatment-naïve melanoma treated with ICB (ICB_Riaz2017_PD1 Ipi_Naive) (Figure 6A, second panel). However, the prioritization of the gene signature in the regulation of TME resistance to ICB occurs in the order C3 > C5 > C3AR1 > C5AR1. However, analysis of the gene knockout phenotype from genetic screens revealed that the knockout of C5 is a strong influencer of lymphocyte-mediated tumor killing in colon cancer models (Kearney 2018 NK_10 and Kearney 2018 NK_20), while C3 knockout influences lymphocyte-mediated tumor killing in the melanoma model (Manguso 2017; GVAX+PD1, Figure 6A third panel). Among the three cell types promoting T-cell exclusion, MDSCs and the M2 subtype of TAMs are negatively associated with the expression levels of C3, C5, C5AR1, and C3AR1, while only C3 expression shows a positive association with cancer-associated fibroblasts (Figure 6A, lower panel).

High C3 expression was associated with a good outcome of ICB therapy of PD1, CTLA4, and ACT in melanoma but was associated with worse PD and PDL1 outcomes in GBM and bladder cancer, respectively (Figure 6B). In contrast, high C5 expression was associated with a worse outcome of ICB therapy of PD1, CTLA4, and ACT in melanoma but predicted a good outcome of PD1 therapy in GBM and kidney cancer (Figure 6C). C5AR1 predicted a worse therapy outcome of PD1+CTLA4 in melanoma and of PD1 in GBM and kidney cancer (Figure 6D). Finally, C3AR1 was associated with a better therapy outcome of PDI and CTLA4 in melanoma and a worse outcome of PD1 therapy in GBM cancer (Figure 6E).

## 4. Discussion

The role of genetic aberrations in cancer diagnosis and prognosis has attracted substantial interest in the field of immune-oncology [44,45]. Our analysis of the differential expression between tumor and adjacent normal tissue, and the genetic as well as epigenetic modulation, of the complement components *C3*, *C5*, *C3AR1*, and *C5AR1* not only demonstrates that the complement components are biomarkers of cancer progression and prognosis but also suggests that these complement components are associated with tumor immune infiltration and immune invasion and could be used to predict patients that would benefit from certain therapies in multiple cancer types. Overall, our differential expression analysis suggests tumor context and stage-dependent heterogeneity in the expression of complement components C3, C5, C3AR1, and C5AR1 in different cancer types. However, a common pattern of sub-type expression among the genes was observed in KIRC, LUAD, LUSC, and STAD. This pattern of gene expression is in alignment with the literature demonstrating the context-dependent expression and functions of complement components in different types of cancer [26,46,47,48,49,50,51].

Moreover, our prognostic analysis revealed that the higher expression levels of C3 in COAD, GBM, KIRC, LGG, and LUSC; C3AR1 in GBM, LGG, and COAD; C5 in COAD, KICH, STAD, and UVM; and C5AR1 in THCA tumors are associated with a shorter survival duration of the cohorts. These cancers have been termed ‘aggressive complements’ as high expression levels of genes coding for complement components were found to significantly impact the poor prognosis of the cohort of those cancers [26]. In contrast, a longer survival duration of the cohorts was achieved with higher expression levels of C3 in ACC, MESO, and SKCM; C3AR1 in ACC and SKCM; C5 in LICH, SKCM, and UCEC; and C5AR1 in DLBC. These cancers have been reported to exhibit “a protective complement” as higher expression levels of genes encoding complement components are associated with a good prognosis of the cohort of those cancers [26]. Collectively, C3, C5, C3AR1, and C5AR1 demonstrated context-dependent expression in and a prognostic impact on various cancer types. However, further experimental validation is required to clarify the role of complement activation in cancer progression and prognosis.

Moreover, the enrichment of *C3*, *C5*, *C3AR1*, and *C5AR1* is associated with complement-receptor-mediated signaling pathways, immune-related processes, and the activation of several oncogenic pathways, including the EMT, Hormone ER, PI3K/AKT, RAS/MAPK, RTK, and mTOR signaling pathways. This suggests that complement proteins could mediate the various oncogenic processes and hence serve as attractive targets for cancer therapy. Experimental evidence also supports the role of complement activation in PI3K/AKT/mTOR-mediated and RAS/MAPK-mediated tumor growth [52,53,54]. C3 promotes the EMT-inducing activity of the pro-inflammatory cytokine TNF-a [55]. C3 has also been reported to activate the renal renin–angiotensin system via induction of the EMT of the nephrotubulus in mice [56], while inhibition of C3aR/C5aR attenuated the proliferation of hepatocellular carcinoma via inhibition of the EMT [57]. Although the gene association pathway analysis revealed a similar pattern of activation of these pathways among the complement components, the pathway, gene, and cancer interaction network reflects a high level of heterogeneity in the susceptibility of different cancer types to different types of pathway activation.

Tumor immune infiltration and immune evasion are reported to be correlated with cancer prognoses and therapeutic responses [22,58]. However, two distinct mechanisms of immune evasion have been proposed. First, infiltration of immune cells into tumors may lead to T-cell anergy or dysfunctional T-cell phenotypes [59,60], while immunosuppressive factors may exclude T-cell infiltration in some tumors [61,62,63], promoting the escape of tumor cells from the host immune system, tumor progression, invasion, and metastasis, and therapeutic resistance [60]. Preclinical evidence also indicates that aberrant infiltration of immune cells into normal tissues may also enhance tumor development and progression [64]. Studies indicate that some oncogenic proteins regulate the infiltration of immune cells into tumors; however, our correlation analysis revealed that the expression levels of C3, C3AR1, and C5AR1 were strongly associated with the infiltration of immune cells into tumors across the TCGA cancer types. Genes highly expressed in tumor cells were expected to have positive associations with tumor purity, while those highly expressed in the TME were expected to have negative associations with tumor purity [27]. Our results demonstrate that the expression levels of C3, C3AR1, and C5AR1 were inversely associated with tumor purity. Based on these findings, we propose that these proteins are mainly expressed in the TME rather than in the tumor cells and that the expression levels are a predictor of the infiltration of immune cells from the TME into the tumor tissues, a conclusion supported by evidence from previous transcriptomic and experimental studies [26,65,66,67,68].

Further, our correlation analysis suggests that these associations between the expression levels of C3, C3AR1, and C5AR1 and tumor immune infiltration could possibly enhance tumor immune evasion via dysfunctional T-cell phenotypes. These findings are supported by various experimental reports implicating C3, C3AR1, and C5AR1 in the modulation of anti-tumor immunity and tumor progression [66,67]. C3 activation induced immunosuppressive neutrophil phenotypes that in turn lead to dysfunctional T-cell phenotypes and impede the antitumor immune response [68]. In contrast, C5 expression demonstrated less of a correlation with immune infiltration and correlated positively with the tumor purity, suggesting its confinement to the tumor rather than the immune cells. This contrasting association is supported by a preclinical study that indicated that C3 drives inflammatory carcinogenesis independently of C5 [65].

Although accumulating evidence agrees on the pro-tumoral role of C5, C3, C3AR1, and C5AR1, the immune infiltration is largely controlled by the properties of the tumor cells themselves [69,70]. For instance, Jackson et al. [65] reported that C3 promotes the onset and growth of cutaneous squamous cell carcinomas (cSCCs) independent of C3aR1 and C5, while de Visser et al. reported that tumor immune infiltration and tumorigenesis of HPV-driven SCC are independent of the C3 pathway [71]. In agreement with a previous study, our analysis revealed a context-dependent association between C5, C3, C3AR1, and C5AR1 expression and tumor immune infiltration across different cancer types. These contextual differences could be attributed to the differences in the immune microenvironment’s composition among cancer types [26]. The contextual role of these complements was more pronounced in THYM; unlike other cancers, the expression levels of C5, C3, C3AR1, and C5AR1 were negatively associated with tumor immune infiltration and positively associated with tumor purity (except for C5AR1), suggesting their confinement to THYM tumor cells. Clinical and experimental evidence of the role of the complements in THYM is limited; however, activation of the complement pathway and formation of the membrane attack complex have been specifically implicated in thymoma-associated myasthenia gravis [72,73] and inhibition of the MAC pathway has been suggested to be a promising treatment for thymoma-associated myasthenia gravis [74,75]. Therefore, the inverse association between C5, C3, C3AR1, and C5AR1 and immune infiltration is not surprising since C5b, C6, C7, C8, and several C9 genes are the major components of the MAC and not C5, C3, C3AR1, and C5AR1.

TAMs, MDSCs, and CAF are immunosuppressive cell populations that infiltrate the TME to promote tumor growth and a worse patient prognosis [16]. MDSCs are immunosuppressive myeloid cell types that could inhibit T cells’ function via C5a/C5aR-dependent upregulation of PDL1, resulting in suppression of the antitumor immune response and a worse patient prognosis [76,77,78,79]. C3 has been reported to promote the differentiation of MDSCs via iC3b/C3d in hepatic cells [80]. Experimental evidence also suggests that C3 regulates resistance to the PD-L1 antibody treatment by modulating TAMs [81]. C5a, an active fragment of C5, mediated macrophage polarization via C5a receptor (C5aR) and NF-κB signaling on TAMs [82,83]. However, the association between C3, C5, C3AR1, and C5AR1 expression and tumor infiltration of MDSCs and TAMs was not statistically significant and, in most cases, an inverse association was found. Therefore, our correlation analysis does not support the role of C3, C5, C3AR1, and C5AR1 in regulating tumor infiltration of TAMs and MDSCs and warrants experimental clarification. Our results suggest that *C3*, C5, C3AR1, and *C5AR1* are associated with tumor immune evasion via dysfunctional T-cell phenotypes with a lesser contribution of T-cell exclusion.

The expression of C3 and C5 does not necessarily guarantee the generation of the anaphylatoxins C3a and C5a, summing up the present correlation analyses with the above-cited experimental evidence that strongly suggests that the polarization of TAMs and MDSCs in tumors are not solely dependent on C3, C5, C3AR1, and C5AR1 activation but rather is associated with the activation of individual products of C3 and C5. In support of this conclusion, a previous study indicated that the anaphylatoxins C3a and C5a (products of C3 and C5) are key players in tumor-specific immunity and clinical responses [84].

Previous studies indicate that CNAs represent driver events during immune evasion and tumorigenesis [85,86,87,88,89]. Here, we report the association between the SCNA of the complement components C3, C5, C3AR1, and C5AR1 and dysfunctional T-cell phenotypes. We found a tumor-context-dependent correlation between a high number of CNAs, a high level of expression of the complement components, and the dysfunctional T-cell phenotypes in head and neck, endometrial, colorectal, liver, and breast cancers. Interestingly, these cancers are among the most mutated cancer types with respect to C3, C5, C3AR1, and C5AR1. These findings suggest the role of SCNA in promoting T-cell dysfunction and that tumor immune evasion is associated with the mutation [86], a hypothesis that aligns with the fact that the tumor types in which CNAs play a more profound role in predicting the immune signature are the types with a high mutation level [85].

DNA methylation is a key epigenetic modification in mammalian genomes that plays a pivotal role in regulating gene expression and may serve as a noninvasive biomarker for cancer diagnosis and prognosis [22,90]. The negative association between the methylation and mRNA level of C3, C5, C3aR1, and C5aR1 is expected to be due to the presence of methyl moieties that inhibit gene expression [91]. Gene methylation functions to recruit repressor proteins or inhibit the binding of the transcription factors to DNA [92]. However, the positive correlation between DNA methylation of C5 and C3 and the mRNA expression in some of the cancers suggests the interplay of other regulatory factors other than DNA methylation [93,94].

Genetic alterations play a pivotal role in the regulation of gene expression and cancer progression [90,95,96]. We found that *C3*, C5, C3AR1, and *C5AR1* were frequently mutated (9~41%) in the studied cancer cohorts. These events may be masked by complex patterns of genetic alterations often associated with genetic instability in later disease stages [97]. Interestingly, the SNV of C5 was found to be linked to prolonged survival of the LGG, LUAD, STAD, and UCEC cohorts; C3AR1 mutation prolonged the lifespan of the STAD and UCEC cohorts; and C3 mutations were found to be linked to a shorter survival of the BRCA and LUAD cohorts. This discrepancy suggests that the mutations of complement components regulate the TME and the prognosis of cohorts by different mechanisms.

However, cancer development and progression cannot be attributed to a single gene as co-occurrences of gene alterations are frequently observed and conjoin with the primary genetic driver to promote tumor progression and limit therapeutic responses [98,99]. Therefore, we conducted a gene mutation co-occurrence analysis and found that SNVs of C3, C5, C3AR1, and C5AR1 significantly co-occurred with the mutation of a number of oncogenic proteins in cancers, suggesting that these genes are functional partners associated with the oncogenic role of C3, C5, C3AR1, and C5AR1. Furthermore, we integrated the complement genes with their co-functional partners across all tumors for enrichment analyses and identified the enrichment of several immune and oncogenic-related pathways and biological processes (Table 1). We found that these biological processes and pathways are similar to the signaling pathways earlier identified to be activated by *C3*, C5, C3AR1, and C5AR1 expression (Figure 1E–H), suggesting that the genes whose alterations were found to co-occur with alterations in the complement proteins are possibly involved in common biological processes and pathways.

Immune checkpoint blockade (ICB) in cancer immunotherapy aids the immune system in recognizing and killing cancer cells [100] by targeting the cytotoxic T-lymphocyte-associated protein 4 (CTLA4), programmed death 1 (PD1), and programmed death-ligand 1 (PD-L1). Disappointingly, only one-third of patients responded to immunotherapy in most of the tested cancer types [101]. Identification of the biomarker signature of tumor immune evasion and regulators of sensitivity/resistance to ICB is a serious unmet clinical need [36].

As expected, we found that high expression of C3, C5, C3AR1, and C5AR1 is associated with worse ICB outcomes in melanoma and bladder cancer. In fact, the gene signature of C3, C5, C3AR1, and C5AR1 gave an AUC value greater than 0.5 in 12 out of the 23 ICB sub-cohorts, suggesting it to be a robust predictive biomarker of ICB. Interestingly, this gene signature demonstrated higher prognostic relevance in ICB sub-cohorts than the widely used ICB response biomarkers, including tumor mutation burden, T.Clonality, B.Clonality, and MSI. Our analysis of gene knockout phenotypes from genetic screens suggested that knockdown of C3 and C5 could influence the lymphocyte-mediated tumor killing in melanoma and colon cancer models. In these models, C3-KO-associated CD8+ T-cell-mediated killing was found to be associated with loss of TNF and IFN-γ signaling pathways in mouse cell lines (MC38-Cas9) of colon cancer [102], while C5-KO-associated T-cell killing was found to be correlated with loss of PDL1, Ptpn2, and NF-κB signaling in a melanoma cell line [103]. More biological evidence is required to further understand the role of the complement components C3, C5, C3AR1, and C5AR1 in cancer progression.

Our results suggest the involvement of C3 and C3AR1 in the resistance of human cancer cell lines to small molecules drugs, while the expression levels of C5AR1 and C3AR1 could predict the response to chemotherapy in breast, ovarian, colorectal, and GBM cancer patients. In line with our observation, a preclinical study reported that the activation of C3 leads to PD-L1 antibody resistance in various human cancer cell lines [81]. Activation of the anaphylatoxin products of C3 and C5 and their receptors C3aR and C5aR [104] increases the expression levels and activities of the IL-6 cytokine, which in turn stimulate angiogenesis and drug resistance in cancer patients [104,105]. Collectively, our study demonstrates that the complement component proteins *C3*, *C5*, *C3AR1*, and *C5AR1* are candidate biomarkers for cancer diagnosis, prognosis, and therapy outcomes, and thus serve as attractive targets for strategizing cancer immunotherapy and the response follow-up.

## 5. Conclusions

In conclusion, the complement components *C3*, *C5*, *C3AR1*, and *C5AR1* demonstrated a context-dependent association with tumor immune evasion, prognosis, and therapy response with high implicative value in melanoma, colorectal, brain, breast, stomach, and renal cancer. Thus, they may serve as attractive targets for strategizing cancer therapy and the response follow-up. However, further experimental assays are required to evaluate the potential of these complement components and to further characterize their precise roles in tumorigenesis and therapy response.

## Figures and Tables

**Table 1:** List of cancer types analyzed in this study.

TCGA Code	Cancer Type	Histology	Body Location
ACC	Adrenocortical carcinoma	Carcinoma	Endocrine
BLCA	Bladder urothelial carcinoma	Carcinoma	Genitourinary
BRCA	Breast invasive carcinoma	Carcinoma	Breast
CESC	Cervical squamous cell carcinoma and endocervical adenocarcinoma	Carcinoma	Gynecology
CHOL	Cholangiocarcinoma (bile duct)	Carcinoma	Digestive
COAD	Colon adenocarcinoma	Carcinoma	Digestive
DLBC	Lymphoid neoplasm diffuse large B-cell lymphoma	Lymphoma	Lymphoma
ESCA	Esophageal carcinoma	Carcinoma	Digestive
GBM	Glioblastoma multiforme	Sarcoma	Neurologic
HNSC	Head and neck squamous cell carcinoma	Carcinoma	Head and neck
KICH	Kidney chromophobe	Carcinoma	Genitourinary
KIRC	Kidney renal clear cell carcinoma	Carcinoma	Genitourinary
KIRP	Kidney renal papillary cell carcinoma	Carcinoma	Genitourinary
LAML	Acute myeloid leukemia	Leukemia	Hematologic
LGG	Brain lower grade glioma	Sarcoma	Neurologic
LIHC	Liver hepatocellular carcinoma	Carcinoma	Digestive
LUAD	Lung adenocarcinoma	Carcinoma	Respiratory
LUSC	Lung squamous cell carcinoma	Carcinoma	Respiratory
OV	Ovarian serous cystadenocarcinoma	Carcinoma	Gynecology
PAAD	Pancreatic adenocarcinoma	Carcinoma	Digestive
PCPG	Pheochromocytoma and paraganglioma (adrenal gland)		Endocrine
PRAD	Prostate adenocarcinoma	Carcinoma	Genitourinary
READ	Rectum adenocarcinoma	Carcinoma	Digestive
SARC	Sarcoma	Sarcoma	Gynecology
SKCM	Skin cutaneous melanoma		Skin
STAD	Stomach adenocarcinoma	Carcinoma	Digestive
TGCT	Testicular germ cell tumors	Carcinoma	Genitourinary
THCA	Thyroid carcinoma	Carcinoma	Endocrine
THYM	Thymoma	Lymphoma	Respiratory
UCEC	Uterine corpus endometrial carcinoma	Carcinoma	Gynecology
UCS	Uterine carcinosarcoma	Mixed type	Gynecology
UVM	Uveal melanoma	Carcinoma	Eye

**Table 2:** Enriched pathways, gene ontologies, and diseases associated with *C3/C5/C3AR1/C5AR1* SNV co-occurring genes.

H.	ID	Name	*p*-Value	FDR B&H	FDR B&Y	Bonferroni
**GO:Molecular Function**	GO:0004875	complement receptor activity	8.290 × 10^−8^	1.981 × 10^−5^	1.200 × 10^−4^	1.981 × 10^−5^
	GO:0004878	complement component C5a receptor activity	4.219 × 10^−6^	3.361 × 10^−4^	2.036 × 10^−3^	1.008 × 10^−3^
	GO:0001856	complement component C5a binding	4.219 × 10^−6^	3.361 × 10^−4^	2.036 × 10^−3^	1.008 × 10^−3^
	GO:0001848	complement binding	2.091 × 10^−5^	1.249 × 10^−3^	7.566 × 10^−3^	4.998 × 10^−3^
	GO:0001847	opsonin receptor activity	4.194 × 10^−5^	2.005 × 10^−3^	1.214 × 10^−2^	1.002 × 10^−2^
	**ID**	**Name**	***p*-Value**	**FDR B&H**	**FDR B&Y**	**Bonferroni**
**GO: Biological Process**	GO:0010575	positive regulation of vascular endothelial growth factor production	3.378 × 10^−7^	5.327 × 10^−4^	4.230 × 10^−3^	5.327 × 10^−4^
	GO:0002430	complement receptor-mediated signaling pathway	2.205 × 10^−6^	8.695 × 10^−4^	6.905 × 10^−3^	3.478 × 10^−3^
	GO:0030449	regulation of complement activation	3.684 × 10^−6^	1.064 × 10^−3^	8.449 × 10^−3^	5.809 × 10^−3^
	GO:0038178	complement component C5a signaling pathway	4.048 × 10^−6^	1.064 × 10^−3^	8.449 × 10^−3^	6.384 × 10^−3^
	GO:0002688	regulation of leukocyte chemotaxis	4.911 × 10^−6^	1.106 × 10^−3^	8.786 × 10^−3^	7.745 × 10^−3^
	GO:0002920	regulation of humoral immune response	8.615 × 10^−6^	1.601 × 10^−3^	1.271 × 10^−2^	1.359 × 10^−2^
	GO:0010758	regulation of macrophage chemotaxis	3.723 × 10^−5^	5.338 × 10^−3^	4.239 × 10^−2^	5.872 × 10^−2^
	GO:0090022	regulation of neutrophil chemotaxis	4.479 × 10^−5^	5.887 × 10^−3^	4.675 × 10^−2^	7.064 × 10^−2^
	GO:0030593	neutrophil chemotaxis	8.376 × 10^−5^	7.812 × 10^−3^	6.204 × 10^−2^	1.321 × 10^−1^
	GO:0002685	regulation of leukocyte migration	8.422 × 10^−5^	7.812 × 10^−3^	6.204 × 10^−2^	1.328 × 10^−1^
	GO:0097529	myeloid leukocyte migration	1.101 × 10^−4^	8.171 × 10^−3^	6.488 × 10^−2^	1.736 × 10^−1^
	GO:1905521	regulation of macrophage migration	1.117 × 10^−4^	8.171 × 10^−3^	6.488 × 10^−2^	1.762 × 10^−1^
	GO:0002253	activation of immune response	1.162 × 10^−4^	8.171 × 10^−3^	6.488 × 10^−2^	1.832 × 10^−1^
	**ID**	**Name**	***p*-Value**	**FDR B&H**	**FDR B&Y**	**Bonferroni**
**Pathway**	1269250	Regulation of Complement cascade	3.005 × 10^−9^	1.337 × 10^−6^	8.929 × 10^−6^	1.337 × 10^−6^
	1269241	Complement cascade	1.130 × 10^−7^	2.513 × 10^−5^	1.678 × 10^−4^	5.027 × 10^−5^
	172846	*Staphylococcus aureus* infection	6.804 × 10^−6^	1.009 × 10^−3^	6.739 × 10^−3^	3.028 × 10^−3^
	M16894	Complement and coagulation cascades	1.570 × 10^−5^	1.747 × 10^−3^	1.166 × 10^−2^	6.986 × 10^−3^
	83073	Complement and coagulation cascades	2.687 × 10^−5^	2.391 × 10^−3^	1.597 × 10^−2^	1.196 × 10^−2^
	1269546	Peptide ligand-binding receptors	5.391 × 10^−5^	3.999 × 10^−3^	2.670 × 10^−2^	2.399 × 10^−2^
	1269203	Innate Immune System	6.409 × 10^−5^	4.075 × 10^−3^	2.720 × 10^−2^	2.852 × 10^−2^
	1269248	Activation of C3 and C5	1.007 × 10^−4^	5.603 × 10^−3^	3.741 × 10^−2^	4.483 × 10^−2^
	M22072	Alternative Complement Pathway	1.722 × 10^−4^	8.515 × 10^−3^	5.685 × 10^−2^	7.663 × 10^−2^
	**ID**	**Name**	***p*-Value**	**FDR B&H**	**FDR B&Y**	**Bonferroni**
**Diseases**	C0025306	Meningococcemia	4.257 × 10^−8^	5.364 × 10^−5^	4.139 × 10^−4^	5.364 × 10^−5^
	C2931788	Atypical Hemolytic Uremic Syndrome	2.466 × 10^−6^	1.554 × 10^−3^	1.199 × 10^−2^	3.107 × 10^−3^
	C0020951	Immune Complex Diseases	6.637 × 10^−5^	1.394 × 10^−2^	1.076 × 10^−1^	8.363 × 10^−2^
	C0003907	Arthus Reaction	1.417 × 10^−4^	1.984 × 10^−2^	1.531 × 10^−1^	1.786 × 10^−1^
	C2717961	Thrombotic Microangiopathies	1.729 × 10^−4^	2.001 × 10^−2^	1.544 × 10^−1^	2.178 × 10^−1^
	C0740345	Germ Cell Cancer	3.289 × 10^−4^	2.072 × 10^−2^	1.599 × 10^−1^	4.144 × 10^−1^
	C0027654	Embryonal Neoplasm	3.289 × 10^−4^	2.072 × 10^−2^	1.599 × 10^−1^	4.144 × 10^−1^
	C0027658	Neoplasms, Germ Cell, and Embryonal	3.289 × 10^−4^	2.072 × 10^−2^	1.599 × 10^−1^	4.144 × 10^−1^
	C0751364	Cancer, Embryonal	3.289 × 10^−4^	2.072 × 10^−2^	1.599 × 10^−1^	4.144 × 10^−1^
	C0003257	Antibody Deficiency Syndrome	4.250 × 10^−4^	2.550 × 10^−2^	1.968 × 10^−1^	5.355 × 10^−1^
	C0008149	Chlamydia Infections	5.355 × 10^−4^	2.550 × 10^−2^	1.968 × 10^−1^	6.747 × 10^−1^
	C0021051	Immunologic Deficiency Syndromes	7.852 × 10^−4^	2.550 × 10^−2^	1.968 × 10^−1^	9.894 × 10^−1^
	C1319860	Sendai virus infection	9.290 × 10^−4^	2.550 × 10^−2^	1.968 × 10^−1^	1.000
	C0221238	Mesangial proliferative glomerulonephritis	1.252 × 10^−3^	2.550 × 10^−2^	1.968 × 10^−1^	1.000
	C0272242	Complement deficiency disease	1.620 × 10^−3^	2.550 × 10^−2^	1.968 × 10^−1^	1.000

## Data Availability

The raw data supporting the conclusions of this article will be made available upon reasonable request.

## Supplementary Materials

The following supplementary materials are available at https://www.mdpi.com/2072-6694/13/16/4124/s1. Table S1: Survival differences between cohorts expressing high vs. low gene expression levels of C3, C5, C5AR1, and C3AR1 across various cancer types, Table S2: C3, C5, C5AR1, and C3AR1 expression correlation with infiltration levels of tumor-associated macrophages (M2-TAMs) across various cancer types, Table S3: Distribution of effective mutations and non-effective mutations of C3, C5, C5AR1, and C3AR1 across various cancer types, Table S4: Gene alterations associated with C3/C5/C3AR1/C5AR1 SNVs, Figure S1: Kaplan–Meier plot of the progression-free survival of cohorts with mutant or wild-type C5 and C3, Figure S2: Heatmap showing the C3, C5, C3AR1, and C5AR1 mutation co-occurrence pattern with other oncogenic proteins, File S1: The individual patient and sample IDs of each TCGA cancer type, File S2: The raw file of the immune infiltration estimation for all TCGA tumor types used in this study, File S3: ImmuCellAI-based correlation between the *C3*, *C5*, *C3AR1*, and *C5AR1* expression levels and the abundance of the immune cells across the TCGA cancer types.
