# Supplementary material for: Pan-Cancer Analysis of Immune Complement Signature C3/C5/C3AR1/C5AR1 in Association with Tumor Immune Evasion and Therapy Resistance
Source: Cancers (Basel). 2021 Aug 16;13(16):4124. doi: 10.3390/cancers13164124 (PMC8394789; doi:10.3390/cancers13164124)
Supplement: Supplementary file 1 [file cancers-13-04124-s001.zip › cancers-1286954-supplementary final.pdf]

Supplementary Materials: Pan-Cancer Analysis of Immune Complement Signature C3/C5/C3AR1/C5AR1 in Association with Tumor Immune Evasion and Therapy Resistance

Bashir Lawal, Sung-Hui Tseng, Janet Olayemi Olugbodi, Sitthichai Iamsaard, Omotayo B. Ilesanmi, Mohamed H. Mahmoud, Sahar H. Ahmed, Gaber El-Saber Batiha and Alexander T.H. Wu

Table S1. Survival differences between cohorts expressing high vs. low gene expression levels of C3, C5, C5AR1, and C3AR1 across various cancer types.

cancertype	symbol	sur_type	hr_categorical(H/L)	coxp_categorical	logrankp	higher_risk_of_death
ACC	C3	OS	0.210134	0.000969	0.000294	Lower expr.
ACC	C3	PFS	0.218855	1.77E−05	3.39E−06	Lower expr.
COAD	C3	PFS	1.786286	0.007853	0.007086	Higher expr.
GBM	C3	PFS	1.409067	0.041031	0.040621	Higher expr.
KIRC	C3	OS	1.472483	0.014077	0.013514	Higher expr.
KIRC	C3	PFS	1.450014	0.017903	0.017287	Higher expr.
LGG	C3	OS	2.141746	0.001053	0.000798	Higher expr.
LGG	C3	PFS	2.303414	3.05E−05	1.81E−05	Higher expr.
LUSC	C3	OS	1.471853	0.023173	0.022331	Higher expr.
LUSC	C3	PFS	1.451787	0.027011	0.026143	Higher expr.
MESO	C3	OS	0.327308	0.044879	0.036307	Lower expr.
SKCM	C3	OS	0.718723	0.04137	0.040435	Lower expr.
ACC	C3AR1	PFS	0.486171	0.025741	0.023173	Lower expr.
GBM	C3AR1	PFS	1.451324	0.026616	0.025507	Higher expr.
LGG	C3AR1	OS	1.641731	0.029289	0.027984	Higher expr.
LGG	C3AR1	PFS	1.674732	0.008073	0.007493	Higher expr.
SKCM	C3AR1	OS	0.607509	0.002085	0.001874	Lower expr.
UVM	C3AR1	OS	6.413163	0.027837	0.014368	Higher expr.
COAD	C5	OS	1.779796	0.038872	0.036289	Higher expr.
COAD	C5	PFS	1.53912	0.048143	0.046684	Higher expr.
KICH	C5	PFS	4.73855	0.049399	0.030221	Higher expr.
LIHC	C5	PFS	0.625433	0.0187	0.017847	Lower expr.
SKCM	C5	OS	0.679179	0.017158	0.016484	Lower expr.
STAD	C5	PFS	1.543903	0.038934	0.03742	Higher expr.
UCEC	C5	OS	0.2765	0.02812	0.018924	Lower expr.
UVM	C5	OS	5.310533	0.03511	0.018477	Higher expr.
DLBC	C5AR1	OS	3.28E−10	0.999175	0.011569	Lower expr.
DLBC	C5AR1	PFS	3.68E−10	0.9989	0.001249	Lower expr.
THCA	C5AR1	PFS	2.314689	0.017261	0.014313	Higher expr.

Table S2. C3, C5, C5AR1, and C3AR1 expression correlation with infiltration levels of tumor associated macrophages (M2-TAM) across various cancer types.

	**C5_M2-TAM**		**C3_M2-TAM**		**C3AR1_M2-TAM**		**C5AR1_M2-TAM**	
cancer	rho	*p*	rho	*p*			rho	*p*
ACC (*n* = 79)	−0.20737	0.078342	−0.25435	0.029895	−0.20893	0.076073	−0.40531	0.000375
BLCA (*n* = 408)	−0.12642	0.015239	−0.34064	1.89E−11	−0.57176	2.50E−33	−0.49657	2.62E−24
BRCA (*n* = 1100)	0.12805	5.15E−05	−0.33488	1.79E−27	−0.51148	2.30E−67	−0.52509	1.57E−71
BRCA-Basal (*n* = 191)	−0.04429	0.561739	−0.45882	1.93E−10	−0.60033	2.04E−18	−0.47978	2.11E−11
BRCA-Her2 (*n* = 82)	−0.08691	0.467878	−0.39819	0.000532	−0.53327	1.41E−06	−0.44266	9.88E−05
BRCA-LumA (*n* = 568)	0.035416	0.421636	−0.3276	2.13E−14	−0.54407	3.60E−41	−0.53811	3.81E−40
BRCA-LumB (*n* = 219)	−0.0757	0.296669	−0.30266	1.98E−05	−0.51239	3.03E−14	−0.46833	7.38E−12
CESC (*n* = 306)	0.130735	0.029602	−0.20951	0.000448	−0.45862	8.19E−16	−0.17874	0.002832
CHOL (*n* = 36)	−0.15311	0.379878	−0.00363	0.983471	−0.62164	6.72E−05	−0.51199	0.001667
COAD (*n* = 458)	0.007524	0.87986	−0.42801	1.62E−19	−0.64343	8.02E−49	−0.65597	2.64E−51
DLBC (*n* = 48)	0.069317	0.666733	0.273336	0.083777	−0.74902	1.77E−08	−0.6009	3.26E−05
ESCA (*n* = 185)	0.050521	0.500613	−0.16806	0.024129	−0.41869	4.94E−09	−0.42214	3.58E−09
GBM (*n* = 153)	−0.04399	0.609772	−0.57486	2.04E−13	−0.52274	5.66E−11	−0.57502	2.00E−13
HNSC (*n* = 522)	0.089132	0.04816	−0.17299	0.000115	−0.48535	1.93E−30	−0.37862	3.23E−18
HNSC-HPV− (*n* = 422)	0.083081	0.097055	−0.16531	0.000904	−0.50556	2.43E−27	−0.40036	7.87E−17
HNSC-HPV+ (*n* = 98)	0.066118	0.538148	−0.21394	0.044104	−0.41025	6.53E−05	−0.27044	0.010372
KICH (*n* = 66)	−0.04441	0.72539	−0.50673	1.65E−05	−0.61813	4.11E−08	−0.63053	1.81E−08
KIRC (*n* = 533)	−0.05415	0.245889	−0.33954	6.67E−14	−0.48198	3.43E−28	−0.52438	6.30E−34
KIRP (*n* = 290)	−0.17604	0.004567	−0.6061	2.88E−27	−0.4764	5.06E−16	−0.60245	7.06E−27
LGG (*n* = 516)	0.028738	0.530793	−0.62694	1.43E−53	−0.62771	9.75E−54	−0.7021	3.29E−72
LIHC (*n* = 371)	−0.28304	8.90E−08	−0.20201	0.000158	−0.41958	3.82E−16	−0.55967	7.89E−30
LUAD (*n* = 515)	0.126206	0.005011	−0.00918	0.838828	−0.51677	5.20E−35	−0.47743	1.96E−29
LUSC (*n* = 501)	−0.06113	0.182549	−0.3478	5.21E−15	−0.56383	2.23E−41	−0.50097	1.13E−31
MESO (*n* = 87)	0.170799	0.118081	−0.02725	0.804497	−0.40877	0.000103	−0.48979	1.96E−06
OV (*n* = 303)	0.088975	0.161601	−0.38486	3.25E−10	−0.53945	3.34E−20	−0.50334	2.13E−17
PAAD (*n* = 179)	0.08495	0.269281	−0.17884	0.019263	−0.39346	1.02E−07	−0.52139	2.64E−13
PCPG (*n* = 181)	−0.02081	0.789517	−0.20608	0.007541	−0.46094	3.65E−10	−0.73199	2.68E−29
PRAD (*n* = 498)	−0.14594	0.002848	−0.532	9.00E−32	−0.4585	5.15E−23	−0.61835	3.05E−45
READ (*n* = 166)	−0.23106	0.006206	−0.45779	1.46E−08	−0.68833	7.77E−21	−0.64912	5.51E−18
SARC (*n* = 260)	0.030234	0.638391	−0.432	1.63E−12	−0.55655	3.02E−21	−0.47842	2.32E−15
SKCM (*n* = 471)	−0.04167	0.374188	−0.29916	6.67E−11	−0.58124	1.19E−42	−0.41271	3.19E−20
SKCM-Metastasis (*n* = 368)	−0.02465	0.643896	−0.21046	6.59E−05	−0.54261	1.71E−28	−0.34305	3.27E−11
SKCM-Primary (*n* = 103)	0.015662	0.875843	−0.26653	0.006773	−0.54134	4.24E−09	−0.41739	1.27E−05
STAD (*n* = 415)	−0.05545	0.281549	−0.24451	1.45E−06	−0.53677	1.15E−29	−0.53973	4.90E−30
TGCT (*n* = 150)	0.017833	0.830248	−0.27408	0.000782	−0.68119	2.22E−21	−0.63972	2.74E−18
THCA (*n* = 509)	0.013348	0.768665	−0.65686	1.40E−61	−0.68059	1.17E−67	−0.7306	1.46E−82
THYM (*n* = 120)	−0.56268	5.96E−11	−0.27292	0.003167	−0.53643	6.45E−10	−0.62335	1.01E−13
UCEC (*n* = 545)	0.186623	0.001332	−0.40425	6.04E−13	−0.46819	2.28E−17	−0.35834	2.64E−10
UCS (*n* = 57)	0.217094	0.118414	−0.57703	6.10E−06	−0.37941	0.00508	−0.5003	0.000136
UVM (*n* = 80)	−0.10236	0.375706	−0.57139	5.73E−08	−0.56967	6.41E−08	−0.46352	2.18E−05

Table S3. Distribution of effective mutation and non-effective mutations of C3, C5, C5AR1, and C3AR1 across various cancer types.

cancertype	symbol	EffectiveMut	NonEffectiveMut	sample_size	percentage	entrez
ACC	C3	0	1	92	0	718
ACC	C3AR1	1	0	92	1.086957	719
BLCA	C3	17	9	411	4.136253	718
BLCA	C3AR1	4	4	411	0.973236	719
BLCA	C5	9	2	411	2.189781	727
BLCA	C5AR1	1	7	411	0.243309	728
BRCA	C3	11	4	1026	1.072125	718
BRCA	C3AR1	2	2	1026	0.194932	719
BRCA	C5	11	7	1026	1.072125	727
BRCA	C5AR1	5	0	1026	0.487329	728
CESC	C3	8	4	291	2.749141	718
CESC	C3AR1	1	1	291	0.343643	719
CESC	C5	2	2	291	0.687285	727
CESC	C5AR1	4	1	291	1.37457	728
CHOL	C3	2	0	36	5.555556	718
COAD	C3	23	16	407	5.651106	718
COAD	C3AR1	8	5	407	1.965602	719
COAD	C5	19	6	407	4.668305	727
COAD	C5AR1	6	3	407	1.474201	728
DLBC	C3	1	1	37	2.702703	718
ESCA	C3	6	2	185	3.243243	718
ESCA	C3AR1	2	0	185	1.081081	719
ESCA	C5	1	0	185	0.540541	727
ESCA	C5AR1	1	1	185	0.540541	728
GBM	C3	11	5	403	2.729529	718
GBM	C3AR1	2	2	403	0.496278	719
GBM	C5	2	2	403	0.496278	727
GBM	C5AR1	2	1	403	0.496278	728
HNSC	C3	14	4	509	2.750491	718
HNSC	C3AR1	5	5	509	0.982318	719
HNSC	C5	9	2	509	1.768173	727
HNSC	C5AR1	1	2	509	0.196464	728
KICH	C5	1	0	66	1.515152	727
KIRC	C3	4	1	370	1.081081	718
KIRC	C3AR1	1	1	370	0.27027	719
KIRC	C5	2	0	370	0.540541	727
KIRC	C5AR1	1	0	370	0.27027	728
KIRP	C3	1	0	282	0.35461	718
KIRP	C3AR1	3	0	282	1.06383	719
KIRP	C5	1	2	282	0.35461	727
LAML	C3	1	0	85	1.176471	718
LGG	C3	9	4	526	1.711027	718
LGG	C3AR1	1	0	526	0.190114	719
LGG	C5	3	2	526	0.570342	727
LGG	C5AR1	0	1	526	0	728
LIHC	C3	12	7	365	3.287671	718
LIHC	C3AR1	1	0	365	0.273973	719
LIHC	C5	6	1	365	1.643836	727

LUAD	C3	15	4	567	2.645503	718
LUAD	C3AR1	11	5	567	1.940035	719
LUAD	C5	8	3	567	1.410935	727
LUAD	C5AR1	7	2	567	1.234568	728
LUSC	C3	23	6	485	4.742268	718
LUSC	C3AR1	10	1	485	2.061856	719
LUSC	C5	12	2	485	2.474227	727
LUSC	C5AR1	6	3	485	1.237113	728
MESO	C3	0	1	82	0	718
OV	C3	6	0	412	1.456311	718
OV	C3AR1	3	2	412	0.728155	719
OV	C5	6	4	412	1.456311	727
OV	C5AR1	3	0	412	0.728155	728
PAAD	C3	3	1	178	1.685393	718
PAAD	C3AR1	1	0	178	0.561798	719
PAAD	C5	1	1	178	0.561798	727
PAAD	C5AR1	1	1	178	0.561798	728
PCPG	C5	1	0	184	0.543478	727
PRAD	C3	3	2	498	0.60241	718
PRAD	C3AR1	2	0	498	0.401606	719
PRAD	C5	2	3	498	0.401606	727
READ	C3	3	3	149	2.013423	718
READ	C3AR1	6	1	149	4.026846	719
READ	C5	4	1	149	2.684564	727
READ	C5AR1	2	0	149	1.342282	728
SARC	C3	4	1	239	1.67364	718
SARC	C3AR1	1	1	239	0.41841	719
SARC	C5	3	0	239	1.25523	727
SKCM	C3	63	45	468	13.46154	718
SKCM	C3AR1	14	3	468	2.991453	719
SKCM	C5	42	18	468	8.974359	727
SKCM	C5AR1	16	21	468	3.418803	728
STAD	C3	31	8	439	7.061503	718
STAD	C3AR1	9	3	439	2.050114	719
STAD	C5	13	9	439	2.961276	727
STAD	C5AR1	5	1	439	1.138952	728
TGCT	C5	1	0	151	0.662252	727
THCA	C3	1	1	500	0.2	718
UCEC	C3	65	27	531	12.24105	718
UCEC	C3AR1	24	10	531	4.519774	719
UCEC	C5	45	24	531	8.474576	727
UCEC	C5AR1	10	6	531	1.883239	728
UCS	C3	0	1	57	0	718
UCS	C3AR1	1	0	57	1.754386	719
UVM	C3	2	0	80	2.5	718

Table S4. Gene alterations associated with C3/C5/C3AR1/C5AR1 SNV.

**C5**	**Cytoband**	**Altered group**	**Unaltered group**	**Log Ratio**	***p*-Value**	***q*-Value**	**Enriched in**
CDK5RAP2	9q33.2	104 (39.69%)	194 (1.81%)	4.45	2.43E−98	5.83E−94	Altered group
CNTRL	9q33.2	102 (38.93%)	198 (1.85%)	4.39	6.59E−95	7.92E−91	Altered group
PSMD5	9q33.2	67 (25.57%)	43 (0.40%)	5.99	8.91E−83	6.48E−79	Altered group
STRBP	9q33.3	77 (29.39%)	90 (0.84%)	5.13	1.08E−82	6.48E−79	Altered group
CUTALP	9q33.2	49 (18.70%)	0 (0.00%)	>10	3.24E−82	1.56E−78	Altered group
PAPPA	9q33.1	102 (38.93%)	309 (2.89%)	3.75	3.64E−79	1.46E−75	Altered group
PHF19	9q33.2	66 (25.19%)	54 (0.50%)	5.64	2.99E−77	1.03E−73	Altered group
RAB14	9q33.2	60 (22.90%)	32 (0.30%)	6.26	1.24E−76	3.72E−73	Altered group
B3GNT10	9q33.2	47 (17.94%)	2 (0.02%)	9.91	9.48E−76	2.53E−72	Altered group
RABGAP1	9q33.2-q33.3	81 (30.92%)	150 (1.40%)	4.46	2.56E−75	6.15E−72	Altered group
ECPAS	9q31.3	83 (31.68%)	171 (1.60%)	4.31	2.53E−74	5.53E−71	Altered group
C3	Cytoband	Altered group	Unaltered group	Log Ratio	*p*-Value	*q*-Value	Enriched in
INSR	19p13.2	142 (33.81%)	176 (1.67%)	4.34	########	########	Altered group
SH2D3A	19p13.3	105 (25.00%)	59 (0.56%)	5.48	########	########	Altered group
GPR108	19p13.3	98 (23.33%)	47 (0.45%)	5.71	########	########	Altered group
ZNF557	19p13.2	106 (25.24%)	87 (0.83%)	4.93	########	7.69E−98	Altered group
TNFSF14	19p13.3	93 (22.14%)	46 (0.44%)	5.66	########	7.87E−97	Altered group
TRIP10	19p13.3	99 (23.57%)	67 (0.64%)	5.21	1.11E−99	4.46E−96	Altered group
ACSBG2	19p13.3	107 (25.48%)	100 (0.95%)	4.75	9.12E−99	3.13E−95	Altered group
VAV1	19p13.3	118 (28.10%)	155 (1.47%)	4.26	1.21E−97	3.63E−94	Altered group
ADGRE1	19p13.3-p13.2	120 (28.57%)	173 (1.64%)	4.12	5.88E−96	1.57E−92	Altered group
PNPLA6	19p13.2	121 (28.81%)	179 (1.70%)	4.08	7.77E−96	1.87E−92	Altered group
RFX2	19p13.3	102 (24.29%)	91 (0.86%)	4.81	2.84E−95	6.20E−92	Altered group
C3AR1	Cytoband	Altered group	Unaltered group	Log Ratio	*p*-Value	*q*-Value	Enriched in
POU5F1P3	12p13.31	149 (57.53%)	4 (0.04%)	>10	6.28E−259	1.51E−254	Altered group
FAM66C	12p13.31	148 (57.14%)	12 (0.11%)	8.99	9.89E−247	1.19E−242	Altered group
FAM86FP	12p13.31	147 (56.76%)	12 (0.11%)	8.98	8.82E−245	5.30E−241	Altered group
LINC00937	12p13.31	147 (56.76%)	12 (0.11%)	8.98	8.82E−245	5.30E−241	Altered group
CLEC4A	12p13.31	161 (62.16%)	47 (0.44%)	7.14	1.59E−243	7.63E−240	Altered group
FOXJ2	12p13.31	167 (64.48%)	72 (0.67%)	6.58	9.45E−241	3.78E−237	Altered group
NECAP1	12p13.31	155 (59.85%)	42 (0.39%)	7.25	2.86E−235	9.82E−232	Altered group
NANOGNB	12p13.31	144 (55.60%)	22 (0.21%)	8.08	2.74E−229	8.21E−226	Altered group
ZNF705A	12p13.31	159 (61.39%)	72 (0.67%)	6.51	1.19E−225	3.18E−222	Altered group
SLC2A3	12p13.31	162 (62.55%)	100 (0.93%)	6.06	1.67E−218	4.01E−215	Altered group
NANOG	12p13.31	150 (57.92%)	58 (0.54%)	6.74	3.03E−216	6.62E−213	Altered group
C5AR1	Cytoband	Altered group	Unaltered group	Log Ratio	*p*-Value	*q*-Value	Enriched in
INAFM1	19q13.32	45 (38.79%)	4 (0.04%)	>10	1.46E−88	3.50E−84	Altered group
NAPA-AS1	19q13.32-q13.33	41 (35.34%)	6 (0.06%)	9.32	3.55E−78	4.26E−74	Altered group
C5AR2	19q13.32	51 (43.97%)	53 (0.49%)	6.49	4.90E−77	3.93E−73	Altered group
TMEM160	19q13.32	43 (37.07%)	15 (0.14%)	8.07	4.32E−76	2.59E−72	Altered group
CCDC9	19q13.32	52 (44.83%)	73 (0.67%)	6.06	8.79E−74	4.22E−70	Altered group
SAE1	19q13.32	51 (43.97%)	69 (0.64%)	6.11	7.24E−73	2.90E−69	Altered group
NAPA	19q13.32-q13.33	48 (41.38%)	50 (0.46%)	6.49	3.42E−72	1.17E−68	Altered group
BBC3	19q13.32	45 (38.79%)	34 (0.31%)	6.95	1.44E−71	4.32E−68	Altered group
SNORD23	19q13.33	39 (33.62%)	16 (0.15%)	7.83	1.87E−67	4.98E−64	Altered group
NPAS1	19q13.32	46 (39.66%)	77 (0.71%)	5.8	4.29E−62	1.03E−58	Altered group

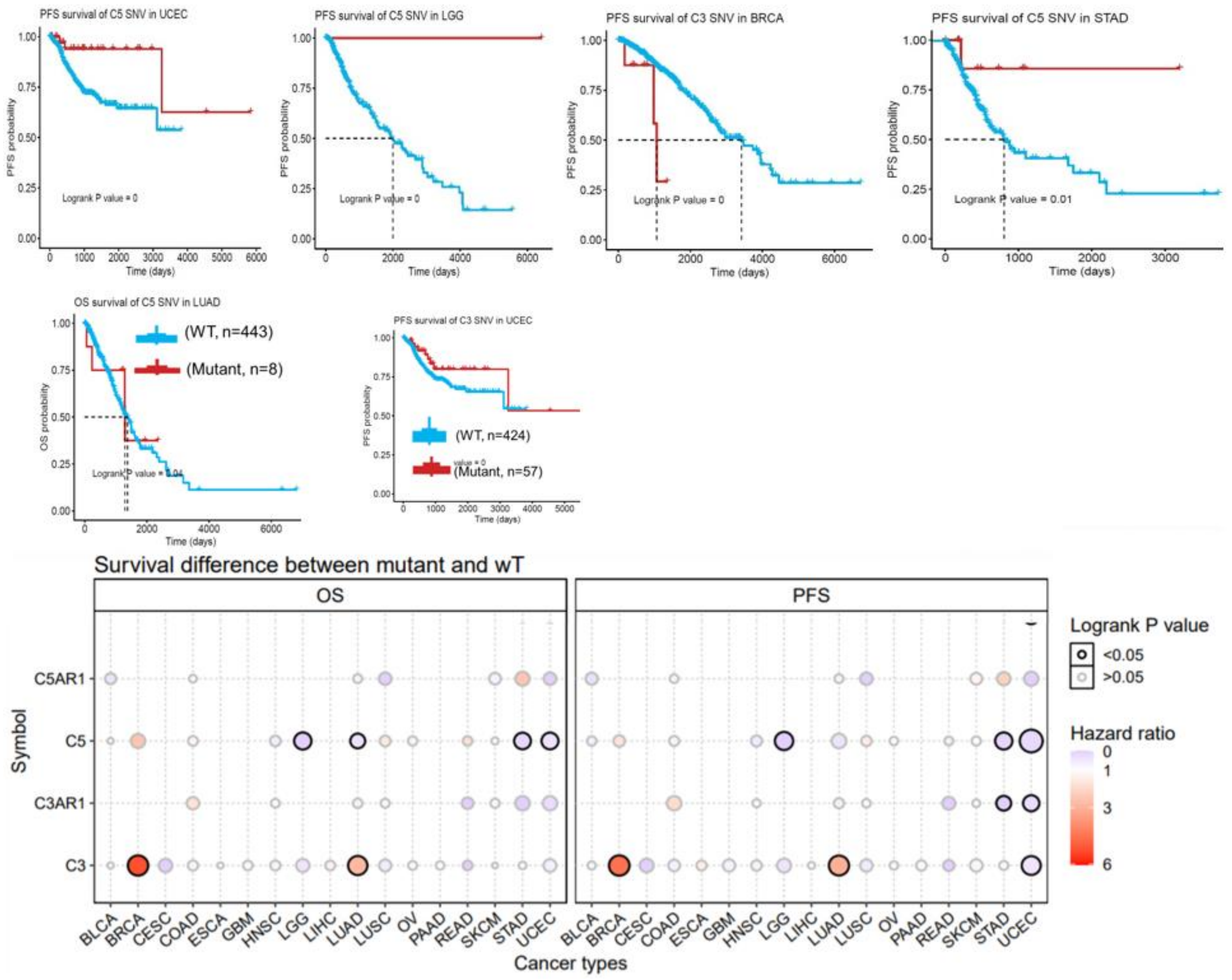

Figure S1. Kaplan Meir plot of the progression-free survival of cohort with mutant or wild type C5, C3, C3AR1, and C5AR1.

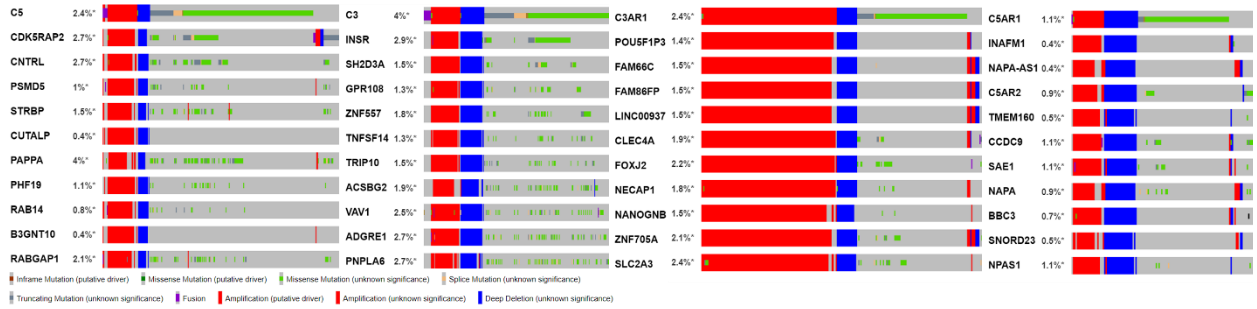

Figure S2. Heatmap showing *C3*, *C5*, *C3AR1*, and *C5AR1* mutation co-occurrence pattern with other oncogenic proteins.

File S1. The individual patient and sample IDs of each TCGA cancer type.

File S2. The raw file of the immune infiltration_estimation for all TCGA tumor types used in this study.

File S3. ImmuCellAI-based correlation between the *C3*, *C5*, *C3AR1*, and *C5AR1* expression levels and the abundance of the immune cells across the TCGA cancer types.

Files S1–S3. are provided separately, attached as Excel files.
